# A Review of Detection and Removal of Raindrops in Automotive Vision Systems

**DOI:** 10.3390/jimaging7030052

**Published:** 2021-03-10

**Authors:** Yazan Hamzeh, Samir A. Rawashdeh

**Affiliations:** 1 Electrical and Computer Engineering, University of Michigan-Dearborn, Dearborn, MI 48128, USA; srawa@umich.edu; 2 Product Development, Ford Motor Company, Dearborn, MI 48126, USA

**Keywords:** raindrop, rain streak, rain detection, de-raining, deep learning

## Abstract

Research on the effect of adverse weather conditions on the performance of vision-based algorithms for automotive tasks has had significant interest. It is generally accepted that adverse weather conditions reduce the quality of captured images and have a detrimental effect on the performance of algorithms that rely on these images. Rain is a common and significant source of image quality degradation. Adherent rain on a vehicle’s windshield in the camera’s field of view causes distortion that affects a wide range of essential automotive perception tasks, such as object recognition, traffic sign recognition, localization, mapping, and other advanced driver assist systems (ADAS) and self-driving features. As rain is a common occurrence and as these systems are safety-critical, algorithm reliability in the presence of rain and potential countermeasures must be well understood. This survey paper describes the main techniques for detecting and removing adherent raindrops from images that accumulate on the protective cover of cameras.

## 1. Introduction

Adverse weather conditions degrade the performance of many image and video-based algorithms used in the automotive domain. Garg and Nayar [1] broadly classify adverse weather conditions into steady (fog, mist, and haze) or dynamic (rain, snow, and hail).

Fog, as an example of adverse weather conditions, reduces the visible range of onboard cameras and causes loss of contrast and fidelity in captured images [2,3]. Rain is a common adverse weather condition and is the focus of this survey paper. In applications that use LIDAR and radar, rain attenuates the strength of the transmitted signals and introduces noise [1]. In image stitching applications, Chia et al. [4] observed that the number of feature points extracted by Harris and SURF detectors was reduced by 48% and 68%, respectively due to falling rain. Barnum et al. [5] used the technique of motion estimation using point trajectories to evaluate the quality of their rain removal algorithm. A 15% to 30% increase in the number of feature points tracked successfully was observed in the de-rained image sequence vs. rainy sequence. Garg and Nayar [6] described a detailed model of falling raindrops, both dynamically (speed, size, shape) and optically (reflection, refraction, warping). More sophisticated models for rendering falling raindrops were later developed (for example, Rousseau and Jolivet [7]) but Garg and Nayar’s model [6] remained the most referenced and used in a good body of research work dealing with falling raindrops and rain streaks detection and removal [8,9]. Tripathi and Mukhopadhyay [10], published a short survey paper on the falling raindrop and rain streak detection and removal algorithms. The focus of that survey was on the different approaches to the detection and removal of falling rain streaks.

This survey paper describes another form of rain, adherent raindrops on the windshield. Due to their irregular shapes, closeness to image sensors, and long temporal presence in captured image frames, adherent raindrop detection and removal problem is relatively harder than that of rain streaks detection and removal [11,12]. Raindrops in these two forms share some common characteristics, such as refraction properties of a raindrop, higher intensity of the drop compared to the background, and average raindrop size. Other characteristics such as raindrop shape and persistence in an image sequence are quite different, which makes models developed for falling raindrops detection and replacement not applicable for adherent raindrops on windshields.

The remainder of this survey paper is organized as follows. Section 2 describes techniques for detecting adherent raindrops and estimating rain intensity. Section 3 describes raindrop models and rendering techniques. In Section 4, adherent raindrop removal techniques for non-automotive applications are described. Section 5 focuses on classical techniques for adherent rain detection and removal, and Section 6 describes detection and removal techniques based on a deep-learning approach. A summary of all techniques discussed can be found in Section 7, and the conclusion is provided in Section 8.

## 2. Raindrop Detection and Rain Intensity Estimation

Adherent raindrop detection algorithms are developed for different application domains, including weather detection and automotive applications. The goal is to classify the weather as either rainy or fair and estimate rain amount/intensity. Raindrop detection can be achieved either by dedicated sensors or general-purpose cameras.

### 2.1. Near-Infrared Sensor Rain Detection

**Figure 1:**
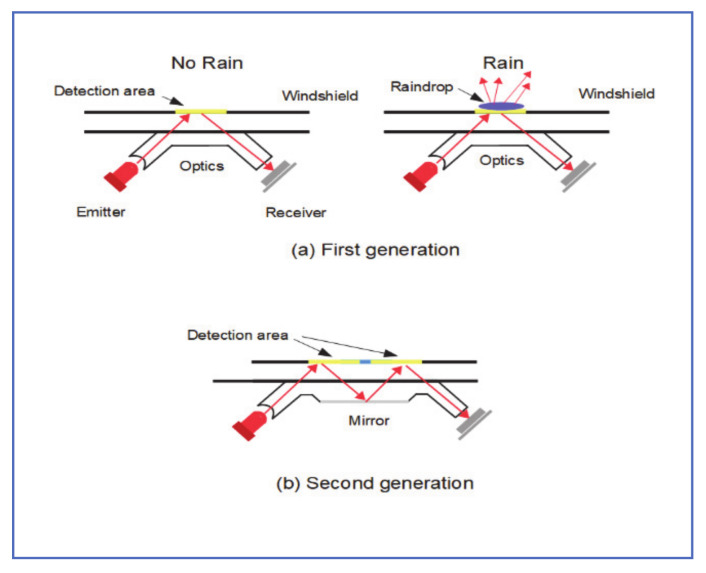
Optical raindrop detection using NIR sensors [13].

As described by Gormer et al. [13], near-infrared (NIR) transmitter and receiver, coupled with optics to enhance performance are commonly used in vehicle rain detections systems. The system measures the amount of light reflected off the windshield area and detects the presence of rain accordingly. To enhance performance, reflective mirrors are added to expand the detection region, as shown in Figure 1. The detection region is still relatively small, and system performance degrades with induced infrared beams from the outside environment.

### 2.2. Camera-Based Rain Detection

**Figure 2:**
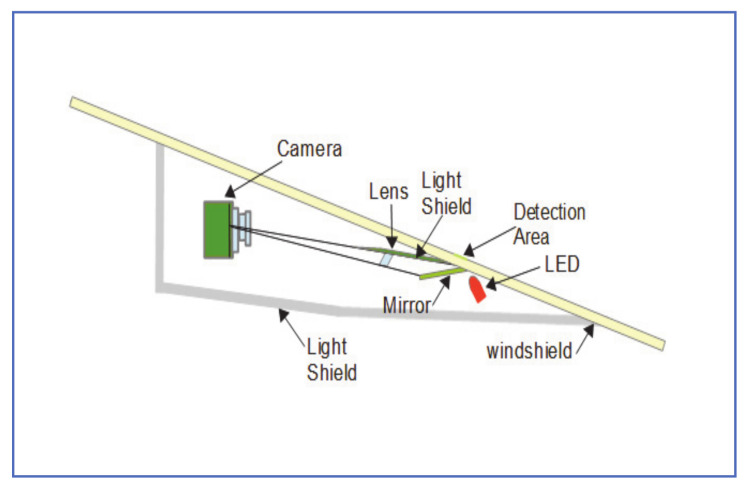
Raindrop detection system using a dedicated camera [13].

As an alternative approach to FIR rain sensors, dedicated camera systems are proposed for rain detection and classification [13,14]. Camera-based rain-sensing provides benefits over FIR-based ones, in terms of improved detection rate, glare reduction, and detection of other particles like dust or salt on the windshield. Gormer et al. [13] have a patent on a camera-based rain detection system, that is also capable of detecting dirt and glare caused by outside light sources. As shown in Figure 2, the system employs an HDR camera with a focusing lens and a light shield to block light outside the detection area. NIR LED is used to provide illumination of the detection area during nighttime or reduced lighting. This system detects raindrops and dirt particles and distinguishes them from more permanent distortions, such as chopped windshield class.

This system is dedicated to raindrop detection and cannot be used for any other image-based applications, such as lane-keeping or traffic sign recognition.

### 2.3. HSV Camera-Based Rain Classifier

**Figure 3:**
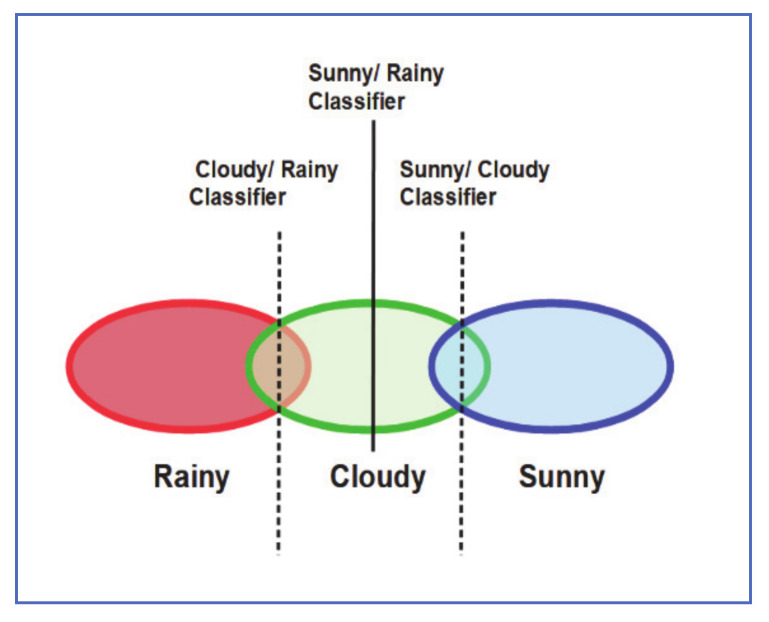
Different weather situations and classifiers [14].

Yan et al. [14] used the histogram of HSV for their proposed weather classification system. Color images are decomposed based on hue, saturation, and brightness, and the histogram of each image is created using 2, 3, 5 bins, respectively. The road surface was chosen as the region of interest and the AdaBoost [15] algorithm was used to combine the weak learners into a strong classifier. Whether classes are distributed along the one-dimensional manifold, as shown in Figure 3. A two-fold classification algorithm is then employed, as described in the process below:

1. Train three different classifiers (Sunny-Rainy, Sunny-Cloudy, and Cloudy-Rainy).
2. Check if the test sample belongs to Sunny-Rainy class.
3. If the sample is classified as Sunny, test it with the Sunny-Cloudy classifier.
4. If the sample is classified as Rainy, pass it with the Cloudy-Rainy classifier, which classifies it as either Cloudy or Rainy.
5. Else, test it with the Cloudy-Rainy classifier, which will classify it as either Cloudy or Rainy, as the final classification result.

For evaluation, Yan et al. tested the performance of their algorithm against other algorithms, namely K-Nearest Neighbor (KNN), AdaBoost with one-vs-all, and AdaBoost.MH classifiers. The algorithm performed better than KNN and AdaBoost.MH, but it was slightly lower than AdaBoost with one-vs-all. In terms of speed, the algorithm was much faster than the others. This algorithm is good for classifying rainy conditions but it is less appropriate for vehicle ADAS applications.

### 2.4. Feature Histogram Rain Classifier

**Figure 4:**
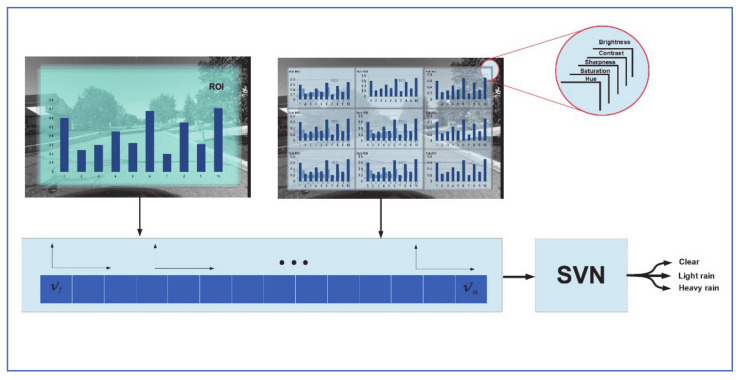
Weather classification using BoF and SVM as described by Roser and Moosmann [16]. The brightness, contrast, sharpness, saturation, and Hue features of an image ROI are used to construct a BoF. And SVN is then used to classify the rainy condition as Clear, Light rain, or Heavy rain.

Roser and Moosmann [16] proposed a weather classification system based on feature histogram and Support vector machine (SVM), using a general-purpose vehicle camera. The output of the system was a classification of the weather as “Clear”, “Light rain” or “Heavy rain”. They selected a bag of features (BoF) that includes brightness, contrast, sharpness, saturation, and hue. The image is defined as the first global Region Of Interest (ROI). It is then divided into 12 sun regions, for a total of 13 ROI per image. Features are measured in each ROI and the output is normalized to a value between 0 and 1, then assigned to one of 10 bins in the feature histogram. The descriptor in this classifier is of size 13 (ROI areas) × 5 (features) × 10 (bins per histogram) = 650 elements. An SVM is added to reduce dimensionality, as shown in Figure 4. To train the system, images were captured under different driving scenarios and labeled based on the weather condition they represented. To test the classifier, three subsets of images were created, each represented one driving scenario (Highway, Highway + Rural, Rural) with different weather conditions equally represented in each subset. Results showed a good rate of classification for the highway-only subset (2% error) but the classification rate got worse as the complexity of the driving environment increased. In addition, for adjacent classes (e.g., “clear” and “light rain” or “Heavy rain” and “light rain”), some misclassification errors were reported. Some background objects were also mistakenly detected as raindrops, which skewed the results of the classifier. Roser and Moosmann’s system does not detect individual raindrops on vehicle windshield which makes it less appropriate for ADAS and autonomous driving applications.

### 2.5. Intensity Gradient-Based Rain Classifier

Cord and Aubert [17] proposed a three-level raindrop classifier (no rain, light rain, heavy rain). They observed that raindrops resemble lenses optically and are almost stationary on a windshield, for a short period. Furthermore, raindrops show high gradient variation have opposite intensity levels (darker on a bright background and vice versa), as compared to no-rain areas of an image. Their algorithm can be summarized as follows:

Six successive images are averaged, to enhance the raindrop signal-to-noise ratio.

The gradient is calculated in the x ($G_{x}$) and y $\left(G_{y}\right)$ directions and total gradient calculated. A Rank filter is applied to the gradient image to create a threshold image.

1- Gradient and threshold images are compared and pixels’ strongest gradients are selected to create a strong gradient image.
2- Regions from the previous step are tested for size range, height-to-width ratio, and eccentricity. Regions that satisfy raindrop threshold ranges are picked as Potential raindrops.
3- Potential raindrops are tracked in the image series and the ones that are detected at least 6 times in 8 consecutive images are identified as raindrops.
4- The gradient amplitude image is calculated as a number between 0 (no rain) to 255 (very high density).

For testing, 2.5 hours’ worth of driving were captured under different rain conditions and driving scenarios. The system showed good results detecting and classifying weather conditions, with minimum misclassifications observed soon after the windshield wiper wipe event. The major issue with the proposed process is that it requires focusing the camera on the windshield to get clear raindrop representation in the captured image.

## 3. Adherent Raindrop Models

In classical image-processing algorithms, main features are identified to model the elements of interest. This section describes some template models for adherent raindrops on windshields and other protective surfaces.

### 3.1. Eigendrops Model

Kurihata et al. [18], observed that a raindrop could be characterized by the outlining edges and refraction of light by the raindrop. They used Principal Component Analysis (PCA) to model adherent raindrops as Eigendrops. The process can be summarized as follows:

1- A total of *K* rectangular sections, each surrounding one raindrop are selected from a windshield image.
2- Rescaling, reshaping, and normalizing processes are applied, to create a one-dimensional unit vector with zero mean: $x_{i}={\left(x_{1},x_{2},\ldots ,x_{N}\right)}^{T}\;\;$
3- A total of *k* randomly selected vectors is used to create the matrix
4- $X=\;\left[x_{1},x_{2},\ldots ,x_{k}\right]$ and its covariance matrix $Q=XX^{T}$ is calculated
5- Eigenvalues are calculated for the covariance matrix and the *r* largest ones are selected and their corresponding eigenvectors calculated $\left\{e_{1},e_{2},..,e_{r}\right\}$, to create the *Eigendrops* subspace.
6- For testing, the Eigenvectors of potential raindrops are calculated and the ones with eigenvectors closest to the *Eigendrops* are identified as actual raindrops.

This model does account for all variations of raindrop shape and size, and the effect of image background on raindrop appearance. Furthermore, to achieve strong and distinctive characteristics that are necessary to create the *Eigendrops*, the camera needs to be focused on the windshield which means it cannot be used for other vision-based automotive applications.

### 3.2. Declivity-Based Model

**Figure 5:**
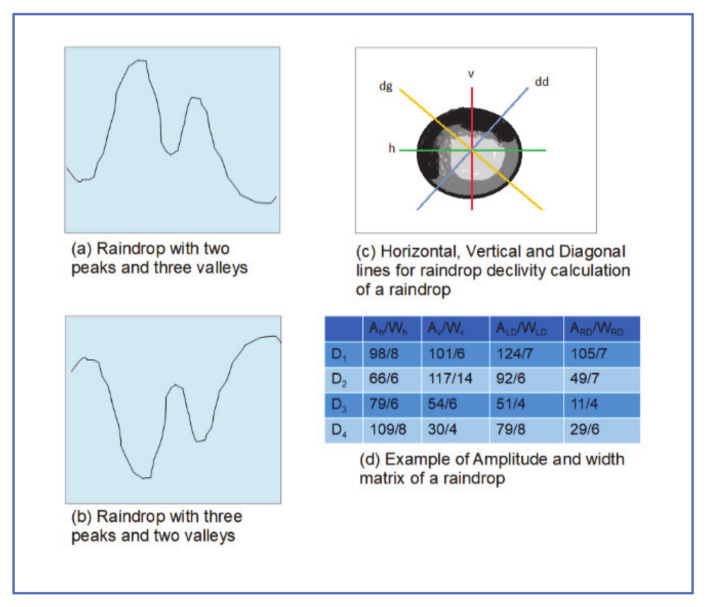
Declivity Method for modeling adherent raindrops [11].

Fouad et al. [11] proposed using a declivity operator to model intensity variations between the adherent raindrop and the background. As shown in Figure 5a,b, adherent raindrop, consists of either two dark regions, separated by light one or two light regions separated by a dark one. The declivity modeling process can be summarized as follows:

1- Gaussian filter is applied on the windshield image with rain, to eliminate noise.
2- Raindrops are selected from the image and declivity calculation is applied in the horizontal, vertical, and two diagonal directions (Figure 5c).
3- The declivity descriptor matrix is constructed from the amplitude and width of each raindrop (Figure 5d).
4- The descriptor is then used to check potential raindrops and identify real ones.

The main issue with this algorithm is that the assumption of the peak-valley appearance of raindrops does not always hold.

### 3.3. Raindrop (RIGSEC) Model

**Figure 6:**
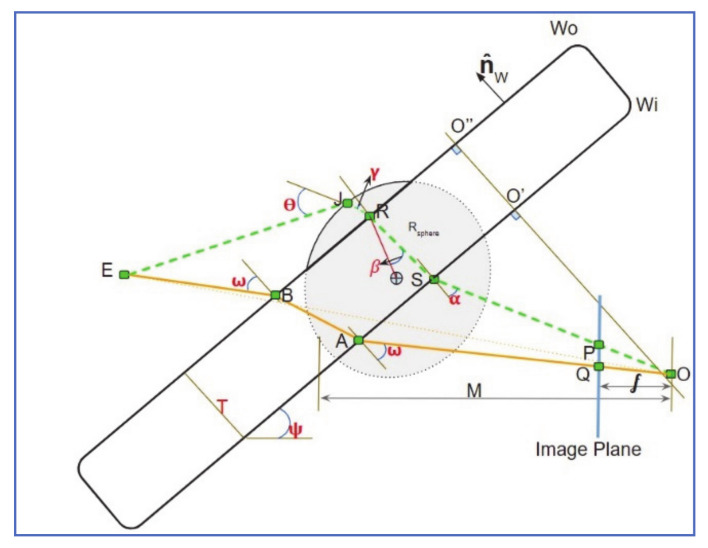
Refraction model of a raindrop on windshield [12]. Each raindrop pixel O in the image plane is traced to the environment point E using lows of light refraction (green line). Numerical calcuations are used to identify the path of the light beam emmitted from point E through the windshield glass only, to the same point O in the image plane (orange line). This ray-tracking method identifies which environment points are captured through the adherent raindrop on a windshield.

Halimeh and Roser [12] developed a geometric-photometric model of raindrops on a windshield, coined Raindrop Intelligent Geometric Scanner and Environment Constructor (RIGSEC). Every raindrop on the windshield act as a convex lens that distorts the background image that surrounds this drop. With a priori knowledge of camera properties and position, and approximation of raindrop shape on the windshield, the distorted image of any section of the image can be modeled. For the geometric part of the *RIGSEC* model, light beams from an arbitrary point E in Figure 6, through different routes; some go through the raindrop and windshield glass (E-J-R-S-P), and others through the glass alone (E-B-A-Q). Using Snell’s law [19], and given the refractors indices of air n_air_, water n_water_ and glass n_glass_, and windshield thickness of T, the through-raindrop and through-glass can be accurately calculated for any point in the camera range of view. A geometric relationship can then be established between distorted P and non-distorted Q projections, and a complete raindrop model can be created at any desired point on the windshield and for any desired raindrop size. To complete the photometric part of the model, Fresnel’s reflectivity equations [20] are then used to calculate the intensity of raindrops as seen by the camera. In raindrop detection applications, many raindrop models are generated with different sizes and positions on the windshield. This establishes a subspace of raindrop templates, that is later used to evaluate potential raindrops.

The *RIGSEC* model is a cohesive, dynamic model, that considers the effect of background variations on raindrop appearance, at different areas of the captured image. It is, however, relatively complex and computationally expensive.

To overcome these issues, Roser and Geiger [21] added some constraints on practical usage of the *RIGSEC* model, in terms of limiting the number of templates generated and restricting the ROI.

### 3.4. Realistic Waterdrops CGI Model

**Figure 7:**
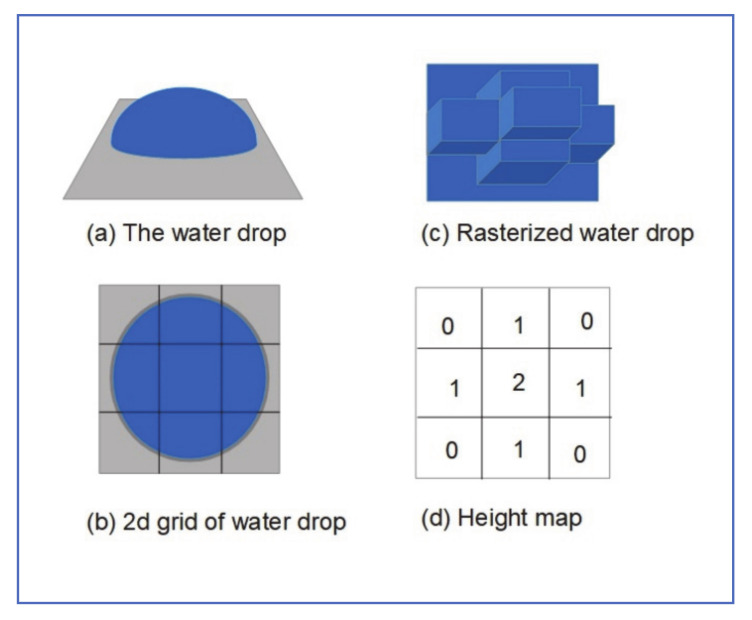
Generating a heightmap for rasterized water drop [22]. A two-dimensional view of the water drop in (**a**) is shown in (**b**). A Rasterized water drop model is generated in (**c**), based on the height map provided in (**d**). The center of the water drop has the largest height value, and the rest take lower height values. No-water areas are reprewsented with height values of zero.

Stuppacher and Supan [22], developed a model for realistic waterdrops, that can be used in game development and CGI, the photometric part of the model was developed as an implementation of the refractions method, described by Tiago Sousa [23]. The authors observed that a certain mass is required for the water drop to move and that small drops stay idle or get swept away by moving drops. In addition, moving drops tend to lose mass, which slows down the drop motion. Waterdrop viscosity, speed, mass, and adhesion to the surface all play a role in shaping the water drop. Water drops are assembled from many small droplets. Waterdrop shape and mass are determined by the arrangement of these droplets. Groups of droplets can split from or combine to moving water drops. Figure 7 shows how water content in each drop can be captured in a heightmap.

Gravitational vector is introduced to the simulation world space, to govern the direction of water movement. Speed and acceleration of water drop on an inclined surface (e.g., windshield) are related to its mass and acceleration. The latter can be expressed in terms of the gravitational vector *G* and inclination angle α (between the surface and the gravitational vector) as:

$$\left(\begin{array}{c} G_{x}^{\prime }\\ G_{y}^{\prime } \end{array}\right)=\left(\begin{array}{c} G_{x}\\ G_{y} \end{array}\right)\cdot \cos\;\left(\alpha \right)$$

To simulate the combining and splitting of raindrops, the “remainder map” and “noise map” were introduced, to control the amount of water lost and gained per water drop.

**Figure 8:**
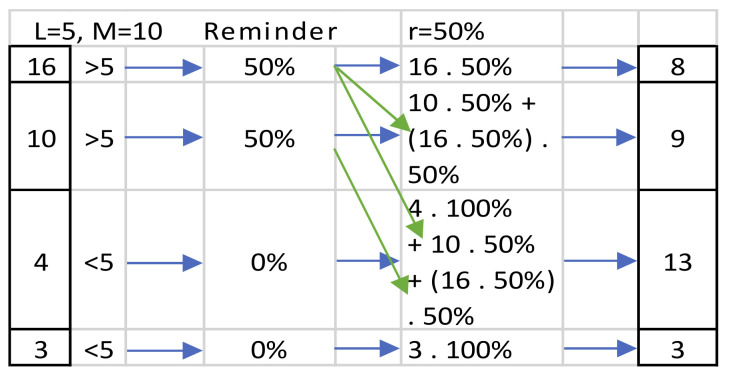
Water mass gain and loss values for reminder and noise factors are set to 50% [22]. The amount of water in a current frame (right-most column) is calcuated from the amount of water in a specific level (left-most column), plus water added from higher levels (blue arrows). The water residue level L=M*Reminder, and the noise level r are used to calcuate the amount of water added.

Figure 8 shows an example calculation of how the waterdrop mass changes while moving down. Water mass at any level is calculated as a sum of excess mass at that level, plus whatever water gained from the level above it.

The model Stuppacher and Supanemploys can be used to generate synthesized datasets in vision-based automotive applications, to augment real datasets.

### 3.5. Bézier Curves Raindrop Model

**Figure 9:**
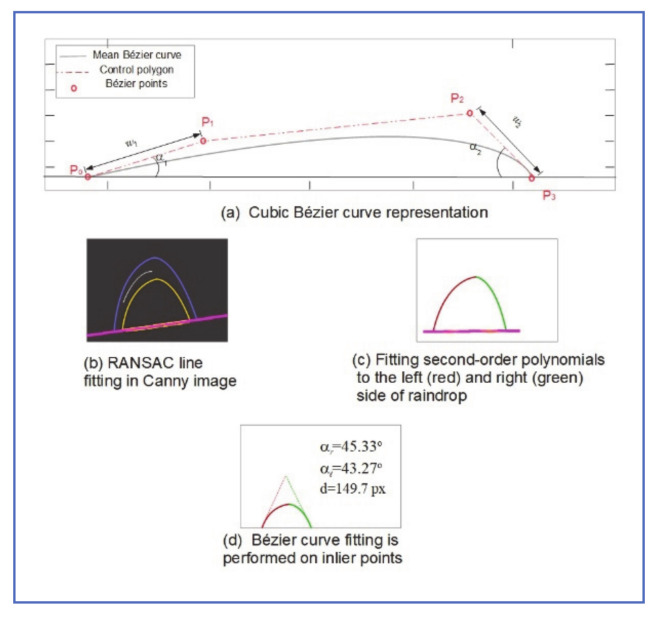
Relating raindrop contact angles and diameter to points of Bézier curve fitted to it [24].

Roser et al. [24], proposed a model for adherent raindrops, using Bézier Curves. An *n*-th order Bézier curve *C* is characterized by a control polygon of *n* + 1 Bézier points ${\left(P_{i}\right)}_{i=0}^{n},\;P\in R^{2}$. It is defined in an interval *t* ∈ [0….1] as:

$$C\left(t\right)\;=\overset{n}{\underset{i=1}{\sum }}B_{i,n}P_{i}$$

where $B_{i,n}\left(t\right)\;=\left(\begin{array}{c} n\\ i \end{array}\right)t^{i}{\left(1-t\right)}^{n-i}$ is the Bernstein polynomial i of degree n. As shown in Figure 9, the Bézier curve can represent the curvature of a raindrop that was deformed from the perfect sphere section, due to gravitational force and plane tilting. Bézier curve parameters (points and weights) are related to the raindrop physical characteristics as follows:

$$\alpha_{1}=∠\left(\bar{P_{0}P_{1}},\bar{P_{0}P_{3}}\right),\;\alpha_{2}=∠\left(\bar{P_{2}P_{3}},\bar{P_{0}P_{3}}\right)$$

$$\omega_{1}=\bar{P_{0}P_{1}},\;\omega_{2}=\bar{P_{2}P_{3}},\;d=\bar{P_{0}P_{3}}$$

where $\alpha_{1}$ and $\alpha_{2}$ are the contact angles of the raindrop, $\omega_{1}$ and $\omega_{2}$ are the Bézier weights, which are related to the physical raindrop centroid shift due to gravity, and the diameter of the raindrop *d* can be used to approximate the raindrop volume.

The authors used two orthogonal Bézier curves to model the raindrop. They used a high-resolution camera, mounted horizontally on a plate that can be tilted, to capture images of different size raindrops and different inclination angles. Canny detector and RANSAC line fitter were used to capture the raindrop edge and mounting surface, as shown in Figure 9b. Next, second-order polynomials were fitted to eliminate more outliers (Figure 9c). Finally, Bézier curve fitting was performed on all inlier points (Figure 9d) and points and weights were estimated. The mean Bézier curve was calculated for all M experiments with different drop sizes and inclination angles. The average Bézier points $\bar{P_{i}}\;$are given by $\bar{P_{i}}=\sum_{k=1}^{M}\frac{P_{i}^{k}}{M}$.

Experimental results showed that it was possible to estimate Bézier curve parameters and model raindrops accurately, just by knowing the raindrop diameter (could be estimated from the SURF algorithm) and the inclination angle of the plate (windshield). The accuracy of the proposed raindrop model was compared to the state of the art, represented in a two-dimensional cut of sphere section, and reductions of up to three orders of magnitude were observed in the Bézier curve-based algorithm as compared to the spherical model.

Despite excellent representation of raindrop shape using this method, practical usage in a real-time image-based application is limited by the accuracy of estimating raindrop volume, which is related to raindrop diameter. This relation is not linear and, when the inclination angle is considered, is not even monotonic.

## 4. Raindrop Removal Techniques for Surveillance Applications

Surveillance cameras are integral components in most security systems. Security in the automotive domain has always been a major concern, the introduction of onboard cameras and specifically 360 degrees camera systems in vehicles [25], 24-h surveillance of vehicle perimeter is now possible. Surveillance cameras may also be mounted on elevated structures, that oversee the surrounding environment, including moving vehicles. Automatic Traffic Monitoring [26] and V2X communications [27] are two applications that use such a setup. This section will describe different raindrop detection and removal methods for camera-based surveillance systems.

### 4.1. Multiple Fixed-Camera Approach

**Figure 10:**
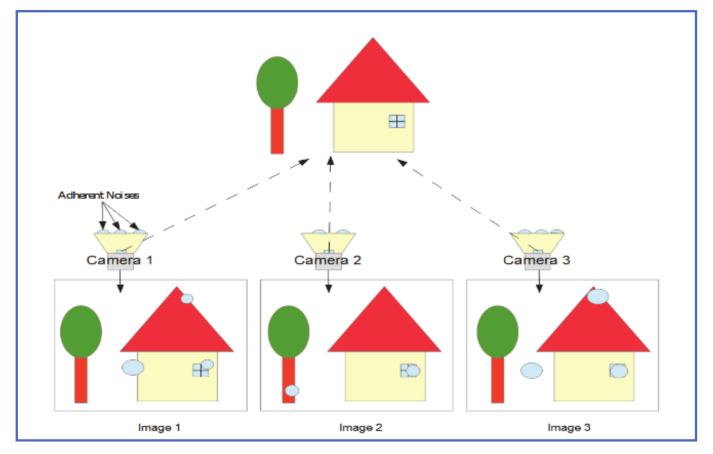
Adherent raindrops are not likely to cover the same areas of an image using different cameras [28].

Yamashita et al. [28], proposed using two or more cameras that are arranged close to each other, to detect raindrops on the camera protective cover (or windshield). As seen in Figure 10, adherent raindrops on images captured by three cameras for the same environment would affect different areas on each image, depending on where raindrops were positioned on each camera cover.

**Figure 11:**
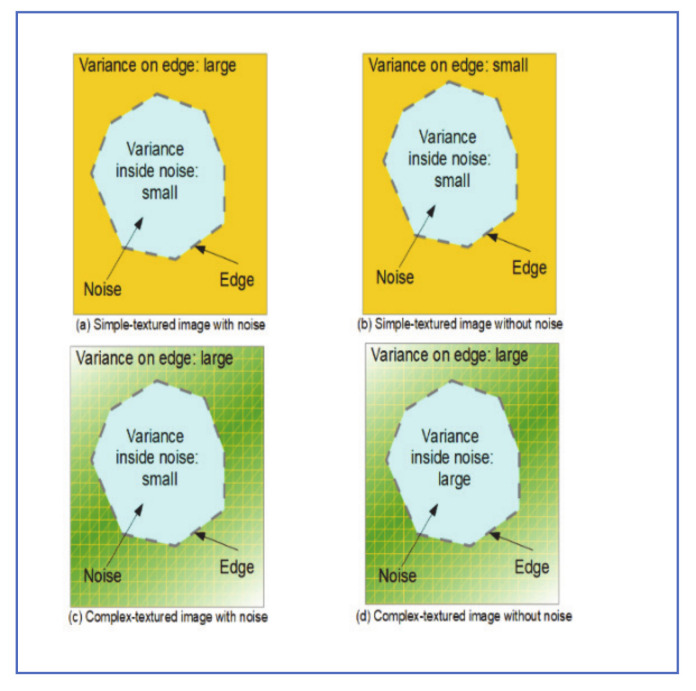
Intensity variance can be used to distinguish raindrop regions from background noise [28].

For a two-camera system, the process can be summarized as follows:

1- Acquire two simultaneous images of the environment and use an algorithm to match the two images.
2- Apply image restoration and chromatic registration on the transformed image, to match the original image.
3- Take the absolute difference in intensity of the two images and assign the “potential raindrops” label to noise patches with an intensity difference greater than a preset threshold. Figure 11 shows the intensity variance for raindrop and background patches, both in a simple and rich texture environment and Table 1 is a truth table that explains the logic of determining raindrop and background regions.
4- Potential raindrops are analyzed to determine true raindrops according to the table below.

To remove raindrop noise, areas of the “clean” image were used to replace raindrop-occluded sections of the other image, after applying the proper restoration and color-corrections.

The system performance was evaluated against other popular image restoration techniques and found to be superior to both inpainting and simple majority approaches. The main drawback of this system is that it assumes that the raindrop-occluded section of an image would be clear in an image taken by another camera. This is not always true, especially under medium-high rain intensities.

### 4.2. Pan-Tilt Camera Approach

**Figure 12:**
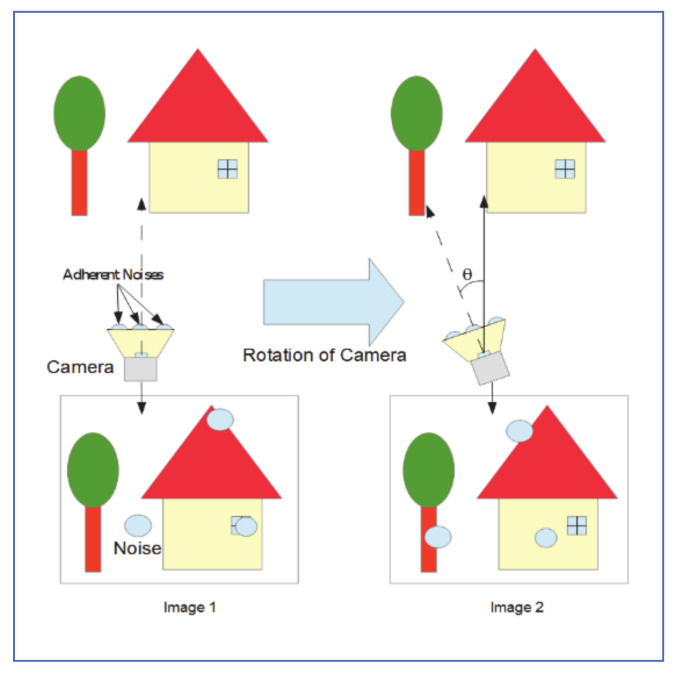
Using a pan-tilt camera for adherent raindrop detection [29].

In another research paper, Yamashita et al. [29], proposed using a pan-tilt camera system, to detect and remove adherent raindrops. As shown in Figure 12, when the camera is rotated, so does the adherent raindrops to its protective cover. This exposes the regions originally covered by raindrops and provides an opportunity for image recovery. The process starts with rectifying radical distortion from the transformed image. Projective transformation and Chromic registration are then applied to match the two images. For potential raindrop noise regions identified in both images, true raindrop must satisfy the maximum distance constraint, as well as size similarity inequality, given by:

$$\frac{min\left(N_{1},N_{2}\right)}{\max\left(N_{1},N_{2}\right)}>U$$

where *N*_1_ and *N*_2_ are the size of the noise region in the original and rotated image, respectively and U is the size similarity factor.

The clean image is finally used to restore raindrop-covered regions of the rainy image.

Test results showed that 92.6% of noises in the original image can be removed. In addition, the process proposed visually outperformed inpainting restoration done with human supervision.

The main drawback of this system is that an accurate measure of rotation angle must be available at all times. Otherwise, all rotational calculations and corrections will be skewed and system performance degrades.

### 4.3. Enhanced Pan-Tilt Camera Approach

Yamashita et al. [30], revisited the pan-tilt approach by using approximated, rather than accurate, angle of rotation in their algorithms.

In addition, rather than using the total size of noise regions, the noise judgment in the enhanced system is based on a pixel-by-pixel comparison. The existence rate is calculated for the potential raindrop regions in the original and rotated images, as $E_{l}=\frac{n_{l}}{N}$, where *N* is the total number of pixels in the noise region and $n_{l}$ is the existence number obtained by incrementing a counter every time a pixel is detected in the same noise region in both images. If the existing rates of the noise are within a given threshold, the region is identified as a real raindrop, otherwise, it is identified as background noise.

Test results showed comparable raindrop detection to the original pan-tilt system that required accurate rotation angle information.

This system still relies on a rotating camera which is acceptable if it is the type of camera used in the surveillance system. If, however, camera rotation is required solely for detection and removing raindrops, then the added cost and complexity may be prohibitive for most automotive applications.

### 4.4. Stereo-Vision Cameras Approach

**Figure 13:**
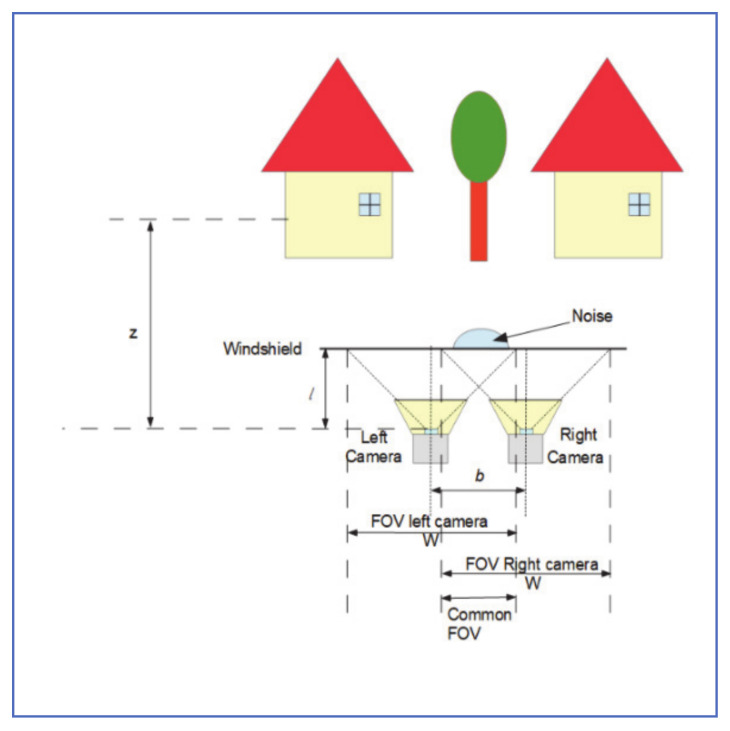
Attributes of a stereo camera system for adherent raindrops detection [31].

Yamashita et al. [31] proposed using a pair of stereo cameras to detect adherent raindrops. As shown in Figure 13, the cameras are set up such that objects in the environment are captured by at least one camera and that the common field of view is large enough.

The process can be summarized as follows:

1- Use normalized cross-correlation (NCC) similarity measure to match the images from both cameras. NCC value *R* is given by:

$$R=\frac{\sum_{j=1}^{N}\sum_{i=1}^{M}\left(I_{l}\left(i,j\right)-\mu_{l}\right)\left(I_{r}\left(i,j\right)-\mu_{r}\right)}{MN\sigma_{l}\sigma_{r}}$$

where$\;I_{lr}\left(i,j\right)$ is the left/right pixel intensity at position $\left(i,j\right)$ in the matching template of size *M* × *N*, $\mu_{lr}$ and $\sigma_{lr}$ are the average and standard deviation of the template pixel intensities, respectively in the left/ right images. Any value *R* less than a threshold *C* is discarded

2- Then, one-on-one matching is applied to the remaining pixels, and the distance between best-matched pixels is used to calculate disparity map S.
3- The disparity of the raindrop pixels on the windshield can be easily calculated by:

$\eta =\frac{bf}{l}$, where *b* is the baseline length, f is the camera focal length and l is the distance between the cameras and the windshield.

4- A pixel at (u, v) is considered a raindrop pixel if $\left|S\left(u,v\right)-\eta \right|<\delta$, where δ is a threshold.
5- The image inpainting algorithm described by Bertalmio et al. [32] was then used to recover rainy segments to form the two images.

Results of visual inspection showed that the algorithm successfully detected and removed raindrops and other adherent noise, both from near and far objects.

### 4.5. Spatio-Temporal Trajectory Approach

**Figure 14:**
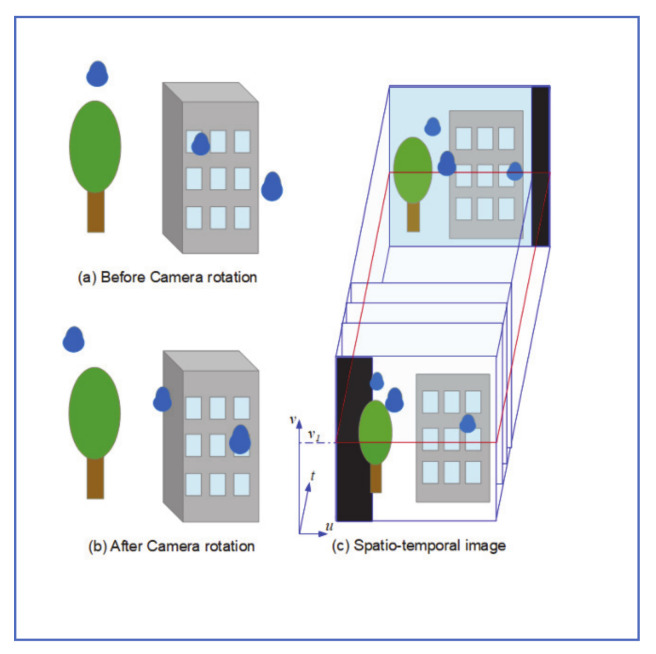
Stacking images in chronological order to generate cross-section image *S*(*u, t*) [33].

Yamashita et al. [33] observed that adherent raindrops on the protective shield of a pan-tilt camera have different trajectories than either static or fast-moving objects in the background. This approach can be summarized as follows:

1- A sequence of images is captured using a pan-tilt camera rotating at a fixed speed.
2- Radial distortion rectification, followed by projective transformation, is applied to the image set.
3- A shown in Figure 14, the captured images are stacked in chronological order, to form the spatiotemporal image *I*(*u, v, t*).

**Figure 15:**
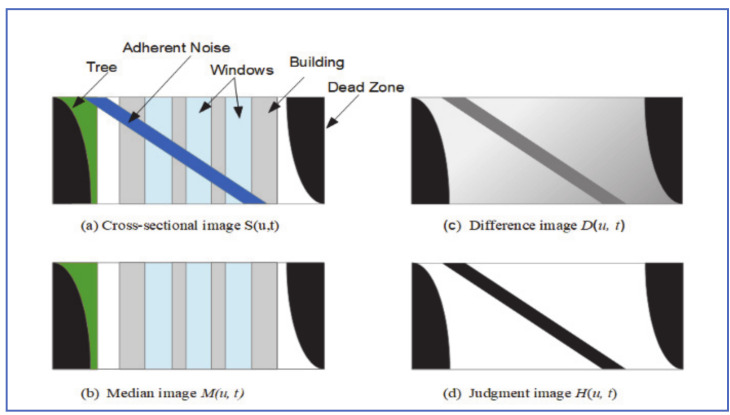
Different images generated in the Spatio-temporal rain detection approach [33].

1- Cross-sectional image *S*(*u, t*) = *I*(*u*, *v*_1_, *t*) is taken of the image stack at level *v*_1_. As shown in Figure 15, trajectories of a stationary object in *S*(*u, t*) are straight lines whereas those of adherent noise are curves.
2- Median image *M*(*u, t*) is then generated which causes adherent noise to disappear, due to its small size compared to other image elements (Figure 15b).
3- Difference image *D*(*u, t*) is then calculated as: *D*(*u, t*) = |*S*(*u, t*) − *M*(*u, t*)|(Figure 15c)
4- Judgment image *H*(*u, t*) that shows candidate noise regions is given by:

$$H\left(u,t\right)=\left\{\begin{array}{c} \begin{array}{cc} 0, & D\left(u,t\right)<T_{b} \end{array}\\ \begin{array}{cc} 1, & D\left(u,t\right)>T_{b} \end{array} \end{array}\right.,$$

where *H*(*u, t*) = 1 for a noise candidate region.

5- The trajectory of each noise candidate curve is tracked in *u* dimension (since *v _=_ v*_1_) and is deemed noise region if the total pixels with *H*(*u, t*) = 1 is greater than a threshold *T_n_*.
6- Steps 4–9 are repeated with new levels *v_i_* until the whole image space is covered. Rainy regions are generated from information gathered from all $H\left(u,t\right)$ images generated.

For rain recovery, Yamashita et al. decomposed the cross-section Spatio-temporal image *S(u, t)* into structure image *f(u, t)* and texture image *g(u, t)*. Inpainting algorithm was applied to structure image, whereas the texture synthesis algorithm described by Efros and Leung [34], was applied to the texture image. The two images were then merged to produce a noise-free image.

Detection results with this algorithm were comparable to those conducted by a human observer. This algorithm requires collecting and post-processing a sequence of image frames over time which makes it not appropriate for real-time automotive applications.

## 5. Raindrop Removal Techniques in the Automotive Domain

In this section, we describe some of the most common algorithms for adherent raindrop detection and raindrop removal (de-raining).

### 5.1. Saliency Maps Approach

Wu et al. [35], proposed an algorithm based on saliency maps of adherent raindrops’ visual features. Saliency maps were created for the raindrop texture, color, and shape features and the AdaBoost algorithm was used to combine the three weak segmenting maps into on strong raindrop detector. The process can be summarized as follows:

1- Color Saliency Map Generation
  - A five-level Gaussian pyramid is created for each color channel (X, Y, and Z) of the image XYZ color map.
  - The center-surround method is implemented between different scales. This produces six difference maps (0,2), (0,3) (0, 4), (1, 3), (1, 4) and (2, 4).
  - An across-scale sum of the six different maps and over all three color-channels is then performed to create a color saliency map.
2- Texture Saliency Map Generation

This process is similar to the color saliency map creation process, with the added step of convolving the image at different scales with the Laplacian of Gaussian (LoG) filter.

3- Shape Saliency Map Generation

Shape feature is extracted on the original image by circle Hough transform (CHT), to generate five accumulator maps, each associated with one raindrop radius value. The shape saliency map is calculated as the sum of the accumulator maps.

**Figure 16:**
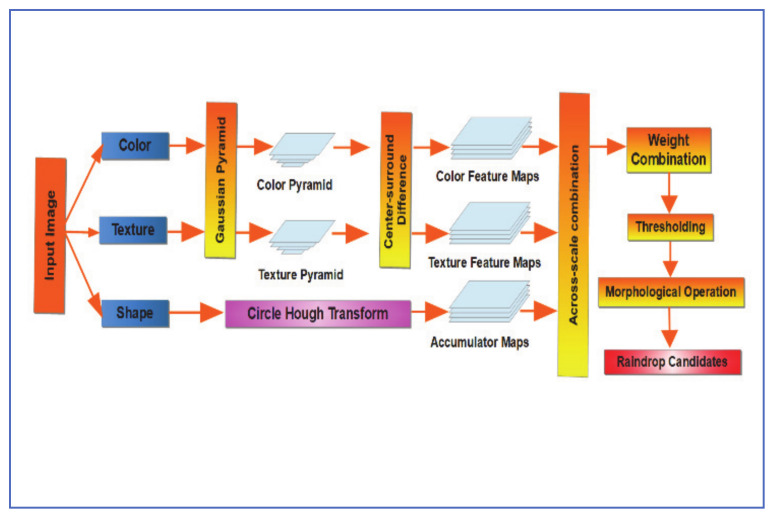
Proposed Raindrop Saliency Map Generation for Raindrop Detection [35].

4- AdaBoost is used to create a raindrop saliency map, from the three different feature saliency maps. Small noise regions are then removed with the help of morphological operations, as shown in Figure 16.

Wu et al. used a digital inpainting technique to remove adherent raindrops from images, by applying smooth propagation in the direction of the lines of equal intensity values (isophones). Precision and Recall metrics were used to compare the performance of this algorithm vs. Kurihata et al. [18] Eigendrops and Roser and Geiger [21] initial raindrop detection stage, done with SURF. Results showed that the proposed algorithm performed better than the other two, both in terms of Precision and Recall metrics. The Proposed algorithm also successfully removed raindrops from images of the windshield at slow driving speed but did not perform as well at higher speeds, due to increased raindrop shape deviation from the assumed circular shape.

### 5.2. Scene Segmentation Approach

Liao et al. [36] divided the scene into the building area and roadway area and used traffic lanes as an indication of the roadway area. Detection of water drops in the roadway section was done using a Sobel edge operator, whereas the histogram equalization method was used in the building section of the image. *N* successive frames were then intersected, to increase raindrop pixel brightness compared to the background. Simple image statistics and adaptive Binary binarization methods were then used to identify potential raindrops and an “or” operator is applied to their results to capture pixels identified in either detection method. Potential raindrops were then compared for shape against elliptical masks of the same size. The remaining potential raindrops were finally checked for average brightness, and the ones brighter than their surroundings were classified as real raindrops.

For rain recovery in the “buildings” zone, an eight-connected areas temple was created around the patch of the water drop and compared with similar areas in the ROI. The region with the highest similarity was used to replace the rain patch. For the road zone of the image with lane marks, a series of dilation and erosion operations were applied to the rain patch area, followed by an image inpainting operation. For non-road mark areas, morphological operations were used for the removal of raindrops.

Test results showed acceptable Peak signal-to-noise ratio (PSNR) on frames with light concentrations of raindrops showed acceptable PSNR (above 30 dB). The detection rate was not affected by raindrop concentration but the removal phase time seemed to increase rapidly with the increased raindrop concentration. The overall processing time was between 732 and 972 ms which might be too long for real-time image-based automotive applications. Another drawback of the proposed technique is that it relied on on-road lane markings and complex building arrangements for image segmentation and raindrop detection. It is not clear how the process would perform with the absence of road marks or lack of buildings. In addition, the process assumed a straight driving scenario, and Liao et al. observed that it was difficult to identify raindrop patches when the vehicle was turning.

### 5.3. Daytime Texture-Based and Nighttime Intensity-Thresholding Algorithms

Ishizuka and Onoguchi [37], proposed two algorithms for raindrop detection; one for daytime and the other for night.

**Daytime approach:** the proposed algorithm can be summarized as follows:

1- An *N* × *M* image is divided into grid blocks *B(u, v)* and Sobel edge detector is used to calculate edge strength *E(u, v)* for each block.
2- *B(u, v)* is classified as a textured block if *E(u, v)* > *T_E_*, where *T_E_* is selected so that the road surface is classified as a non-textured block.
3- Is applied in an image I with two variance values, σ_1_ and σ_2_ (σ_2_ > σ_1_), resulting in two images, $I_{s1}$ and $I_{s2}$.
4- The degree of blur $D_{b}$ is given by:
  $D_{b}=\frac{I_{e1}}{I_{e2}}$, where $I_{e1,2}$ is the edge strength for image $I_{s1,2}$, calculated using a Sobel edge detector.
5- A pixel (*i, j*) is chosen as a raindrop candidate as follows:
  - In a non-textured block, if there exist one or more pixels (*k,* *l*), such that $D_{b}\left(i,j\right)-D_{b}\left(k,l\right)>T_{n}$, where $T_{n}$ is a threshold, then (*i, j*) is a raindrop pixel candidate.
  - In textured block, if there exist one or more pixels (*k,* *l*), such that $D_{b}\left(k,l\right)-D_{b}\left(i,j\right)>T_{t}$, where $T_{t}$ is another threshold, then (*i, j*) is a raindrop pixel candidate.
6- The histogram of optical flow is finally measured over 15 frames of the potential raindrop pixels. If the histogram of optical flow is consistent, then it belongs to the background. If it has various directions, then the pixel belongs to a real raindrop.

**Figure 17:**
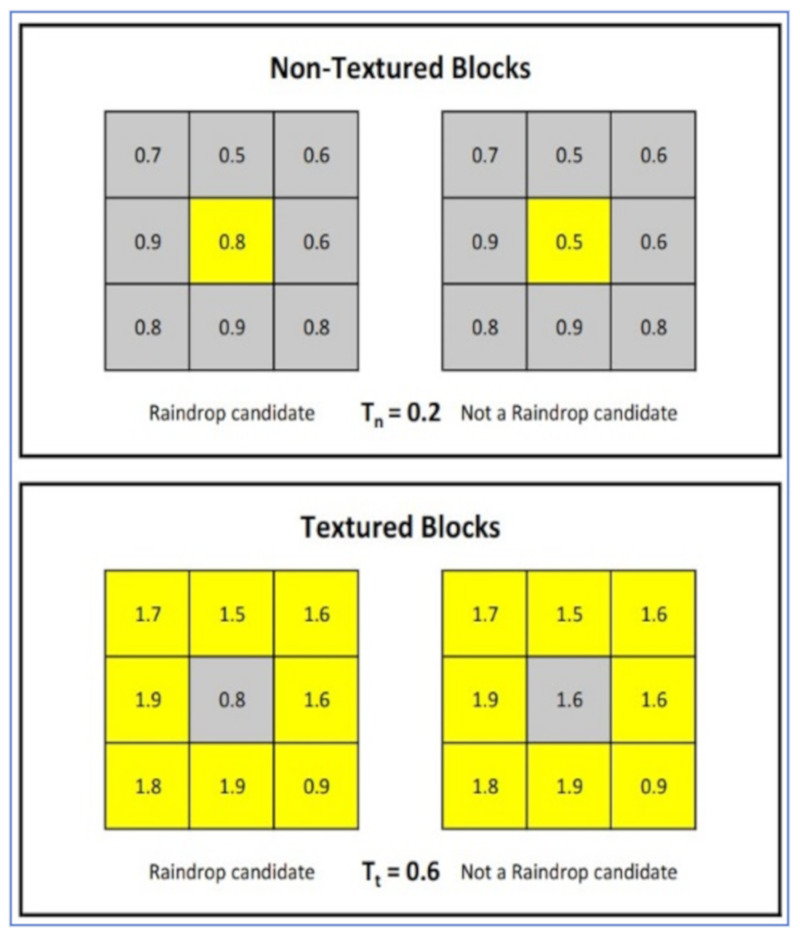
Raindrop candidate pixels are chosen based on the difference between their degree of blur value and those of the neighboring pixels [37].

Figure 17 shows four different scenarios that clarify these classification criteria.

**Nighttime approach:** the process for detecting adherent raindrops at night can be described as follows:

1- Pixels representing light sources and neighboring areas can be estimated by simple binarization and eliminated from the detection process.
2- Differential image $FD_{t}\;$ is generated from images in frames *t* and *t* − 1:
  $$FD_{t}\left(i,j\right)\;=\left\{\begin{array}{cc} 1 & if\;I_{t}>T_{dark}\;and\;\left|I_{t}\left(i,j\right)-I_{t-1}\left(i,j\right)\right|<T_{dif}\\ 0 & otherwise \end{array}\right.$$
  where $T_{dark}$ and $T_{dif}$ are experimental thresholds. This step eliminates pixels that are too dark and defines them as candidate raindrop pixels the ones whose intensity did not change much ($<T_{dif}$).
3- Integration image $SFD_{t}$ is created by adding the last m frames. This image is then and small regions are discarded since they are not likely to represent raindrops. The remaining regions represent adherent raindrops.

At daytime, results showed that most raindrops were correctly detected, but some ones in the sky region were missed. At night, most raindrops were also detected, except the ones around light sources. Processing time was 10 and 30 frames/second for daytime and nighttime raindrop detection, respectively.

### 5.4. Background Subtraction and Watershed Algorithms

Cord and Gimonet [38], proposed an adherent raindrop detection algorithm that uses either background subtraction or a watershed approach for identifying raindrop candidates.

The process can be summarized as follows:

1- Segment the image into bright and dark regions and apply detection on the dark regions only. This is done by applying erosion of the image, followed by morphological reconstruction of the original image with the eroded one. and applying Ostu’s method [39] to segment the image into dark and bright regions.
2- To identify raindrop candidates, one of the two approaches are used:
  - Background Subtraction: For each channel of the RGB image, a large Gaussian filter is applied to eliminate (mask) raindrops. The difference between filtered and original image is then taken which exposes the potential raindrop regions. Morphological operations are then applied and the masks from the three RGB channels are added to the mask in step 1 to produce the candidate raindrop regions.
  - Watershed: Gaussian filtering is applied to the grayscale image then watershed is used to segment the image and extract potential raindrop regions.
3- Raindrop candidates are then compared with an ellipsoid model of similar size, and regions that do not match are eliminated.
4- Temporal information is used to eliminate regions that show up less than two times in the last three successive frames.

**Figure 18:**
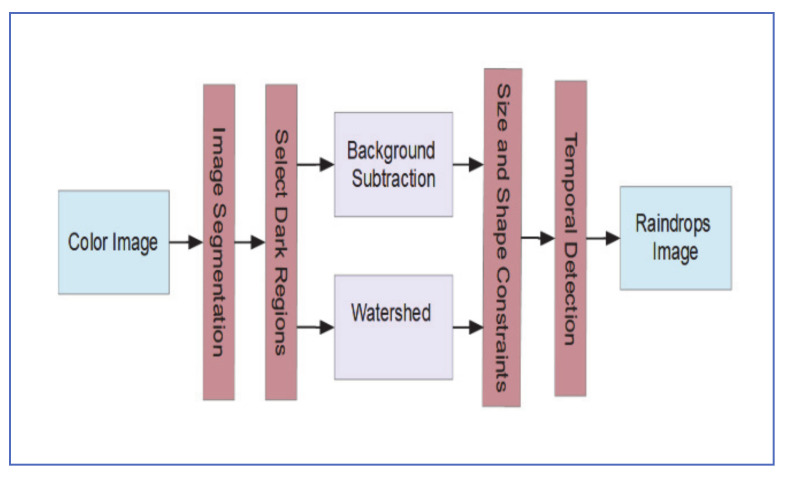
Raindrop detection algorithm described by Cord and Gimonet [38].

Figure 18 shows the main stages of this Algorithm.

The performance was evaluated using three performance metrics. They are:

Correct Detection Rate $CDR=\frac{TP}{P}\;$.

False Positive Per Image $FPPI=\frac{FP}{n}$ and Dice Coefficient $Dice=\frac{2TP}{TP+FP+P\prime }$, where *TP* is the number of true positives, *FP* is the number of false positives, *P* is the total number of raindrops, *n* is the number of images and dice is the size of the union of two sets, raindrops and detection, divided by the average size of the two sets. In terms of raindrop localization, the watershed-based approach was twice as slow as the background subtraction one. Performance-wise, ***CDR*** values were similar for both approaches, but ***FPPI*** and dice showed better performance of watershed compared to background subtraction. In terms of raindrop prediction, ***FFPI*** (a measure of false detection) stayed high, which was attributed to insufficient temporal filtering (3 consecutive frames). Cord and Gimonet predicted that increasing the temporal period to 1 s = 15 frames would improve FPPI. Processing speed was the main drawback of this algorithm. It was, however, coded in MATLAB and run on a relatively slow machine. It would be interesting to see how this algorithm performs on dedicated image-processing hardware and embedded code.

### 5.5. Blurriness-Based Approach

Raindrop images captured with general-purpose cameras tend to be blurry, with very weak boundaries that separate them from their background. Nashashibi et al. [40] used this characteristic to detect adherent raindrops on windshields. The process can be summarized as follows:

1- Image pixels that satisfy the following intensity inequality are chosen as potential rain pixels are extracted through segmentation, using three constraints on pixels intensity and noise region roundness:
  $p_{2}\geq \Delta I=I_{n}-I_{n-1}\geq \;p_{1}$, where $p_{1}$ and $p_{2}$ are chosen to pick the brightest pixels, while reducing the intensity variations introduced by fast-moving objects in the background.
2- Smaller regions are combined with larger ones, using an 8-connectivity extractor. The resultant regions that pass the roundness test below are considered candidate raindrops: $\left|\frac{CC_{Area}^{2}}{CC_{perimeter}}\right|\;\leq 4\pi p_{3}$, where *CC* represents the tested noise region.
3- Contours of candidate raindrops are summed on two consecutive frames and subtracted from the candidate raindrop regions. The Canny filter is used to detect edges of the resultant regions, which now are considered raindrop regions.

**Figure 19:**
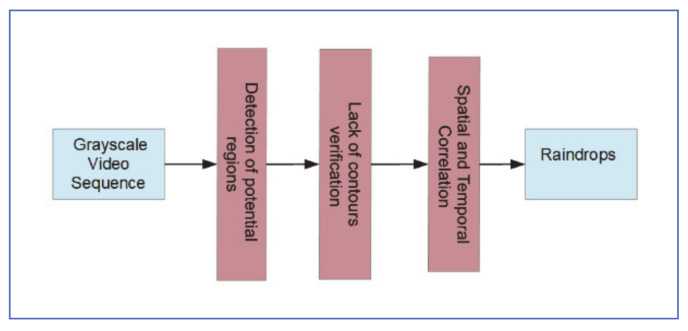
Detection algorithm described in [40].

Figure 19 shows the algorithm stages.

Test results showed that the algorithm correctly detected rain situations as heavy, medium, or no-rain.

Though promising, Nashashibi et al. did not present data to show how accurate the system was in detecting individual raindrops. Rather, the algorithm was tested as an enabler to the rain classifier system which is not as useful in ADAS and autonomous driving applications.

### 5.6. Raindrop Detection Algorithm Using RIGSEC Model

Halimeh and Roser [12] used the RIGSEC model to detect adherent raindrops on special tiltable planes, with artificial patterns and real traffic scenes. The correlation coefficient similarity measure was used to evaluate the observed (real) and estimated (with RIGSEC) pixel intensity. It is given by:

$$CC=\frac{1}{N\sigma_{\hat{I}}\sigma_{I}}\overset{N}{\underset{i=1}{\sum }}\left(\hat{I_{i}}-\bar{\hat{I}}\right)\left(I_{i}-\bar{I}\right)$$

where *N* is the number of all estimates, $\bar{I}$, $\sigma_{I}$ are the average and standard deviation of pixel intensities of observed values, $\bar{\hat{I}}$, $\sigma_{\hat{I}}$ are average and standard deviation of pixel intensities of estimated values. MaxCorr value was defined as the maximum *CC* in a small ROI around the estimated raindrop. Results of artificial pattern and drive scene showed good visual similarity between real and modeled raindrop. Maxcorr results were also good but *CC* results were low. Halimeh and Roser attributed this to the raindrop lens effect and blurriness of raindrop appearance, due to camera focus near infinity. In terms of raindrop detection, the lower performance was attributed to inaccuracies in the initial localization of candidate raindrops, using the SURF algorithm. Further experiments showed that matching the constructed environment within a small ROI, raindrop positions converge to the optimum, and *CC* values converge to MaxCorr values as well.

### 5.7. Raindrop Detection Using Enhanced RIGSEC (fastRIGSEC) Model

Roser and Geiger [21] created an enhanced version of the RIGSEC model named *fastRIGSEC*. First, rather than considering the whole windshield image, the enhanced algorithm runs only on a specific ROI that is required by most vision-based algorithms. In addition, a limited number of raindrop templates are synthesized at an equal distance over a grid that covers the ROI. During the detection phase, these templates are correlated to potential raindrops that fall in their grid cell regions. This enhancement is introduced to improve the performance speed of the algorithm.

Secondly, SURF for identifying potential raindrop was replaced by a more accurate technique, that can be described as follows:

- The original image is bandpass-filtered using the Difference of Gaussian algorithm (DOG).
- The resultant image was segmented and small segments were combined using a connected component algorithm.
- Eigenvalues are calculated and used to filter out non-raindrops, based on convexity ratio, dominant orientation, and aspect ratios of the potential raindrop blobs.
- To identify actual raindrops, the remaining candidates are compared to the artificially created raindrop, using two similarity metrics; intensity Correlation coefficient (CC_intensity_) and Correlation coefficient of their first derivative (CC_gradient_).

**Figure 20:**
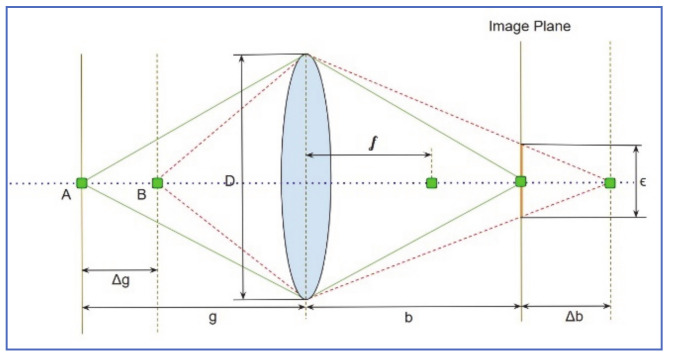
Close objects to the camera are projected as a blurry disk on the image plane [21].

The third enhancement involves adding a level of blurriness to the generated raindrop. As shown in Figure 20, point A that belongs to a far object is projected right on the image plane of the camera. Point B that belongs to nearby objects, however, is projected somewhere behind the image plane. In that sense, Point B projection looks like a desk, rather than a focused pixel on the image plane. The diameter of this disk kernel is given by:

$$\epsilon =\frac{∆gf^{2}}{O\left(g-∆g\right)\left(g-f\right)}$$

where *O* is the camera aperture size, *f* is the focal length, *g* is the distance between the camera lens and far objects, and Δ *g* is the difference in distance between far and near objects.

The introduction of a blurry disk improved the resemblance between modeled raindrop and raindrop projection captured by the camera. For rain recovery, Roser and Geiger developed a reconstruction process, that can be summarized as follows:

1- Define $\Theta_{i}$, a 6D vector that represents rotational and translational parameters between frames i and i+1:

$$\Theta_{i}=\left(r_{x}\left(i\right),r_{y}\left(i\right),r_{z}\left(i\right),t_{x}\left(i\right),t_{y}\left(i\right),t_{z}\left(i\right)\right)$$

The task is to solve for this vector and use it for recovering rainy pixels in one frame from clear ones in another.

2- The road plane is initially wrapped from frame i to i+1, using prior estimates of $\Theta_{i}$ and bilinear interpolation.

3- Harris detector and NCC are then used to match features from the two frames. Direct Linear Transformation (DLT) [41] and RANSAC [42] are then applied to eliminate any outliers.
4- To refine the parameter set $\Theta ={\left\{\Theta_{i}\right\}}_{i=1}^{N-1}$, a MAP solution of the equation below is required (*N* is the total number of frames):
  $$P\left(\Theta |Z_{1},\ldots ,Z_{N}\right)\;\propto P\left(Z_{1},\ldots ,Z_{N}|\Theta \right)P\left(\Theta \right).$$

This assumes independence of non-consecutive frames and normal distribution for the observation probability $P\left(Z_{i}|\Theta_{i}\right)$. Independence over translational and rotational parameters is also assumed which leads to:

$$P\left(\Theta \right)\;=P\left(r_{x}\right)P\left(r_{y}\right)P\left(r_{z}\right)P\left(t_{x}\right)P\left(t_{y}\right)P\left(t_{z}\right)$$

5- After Θ parameters are estimated, the multi-band blending process is used to reconstruct raindrop-covered areas of the image.

Precision and recall measures are used to evaluate the performance *fastRIGSEC* algorithm. The algorithm is compared to SURF, BLUR, and a combination of BLUR and RIGSEC. FastRIGSEC performed better than SURF and BLUR but fall behind (BLUR + RIGSEC) in terms of maximum precision achievable. FastRIGSEC still performed better than the rest in terms of maximum recall rate achievable. In terms of image restoration, *fastRIGSEC* performed well but fall slightly behind (BLUR + RIGSEC), especially with an increase in raindrop density.

### 5.8. Raindrop Detection Using Extended Maximally Stable External Regions

Sugimoto et al. [43] used Extended Maximally Stable External Region (*eMSER*) algorithm to detect potential raindrop blobs. In this approach, raindrops 2D impression is approximated with an ellipse and the 3D shape as a spheroid. Flattening of a spheroid is defined as $f=1-\frac{a_{s}}{a_{l}}$, where $a_{s}\;$and $a_{l}$ are the short and long axes of the ellipse, respectively. The raindrop detection process can be summarized as follows:

1- Candidate raindrops are fitted to the nearest ellipse, using the least square method.
2- Ellipse orientation and long and short axes are measured and flattening values are calculated.
3- Raindrop candidates are identified as real raindrops if their flattening value f satisfied:
  $0<f_{lower}<f<f_{upper}<1$, where $f_{lower}$ and $f_{upper}$ are the lower and upper limits for true raindrop ellipsoid, respectively.

Precision and recall measures were used to compare the detection performance of eMSER against SURF and classical MSER approaches. Of the three evaluated, SURF performed worse and eMSER showed the best results, with rates for (precision, recall) ranging from (0.8, 0.38) to (0.68, 0.5), respectively.

The main drawback of this approach is that it does not describe any mechanism for rainy image restoration. Anyone of many restoration techniques can indeed be used in conjunction with eMSER but, as we described in other approaches, the restoration stage usually is closely integrated with the detection stage, and the overall performance of the system is not a simple multiplication of the performances of each stage.

## 6. Neural Networks and Deep-Learning Techniques

Deep learning and convolutional neural networks (CNN) showed great success in the field of image processing. Unlike classical image processing techniques that operate on the image, features are required lots of processing stages, CNN accepts an image as a whole and “learns” features progressively through its many layers. Image denoising, of which image de-raining is a special case, has been approached by many CNN- based systems, including Gradient-Based learning and Generative Adversarial Network (GAN). Systems based on conventional neural networks (NN) were also proposed for image denoising, such as the Multi-layer perceptron (MLP) [44] and Block-matching and 3D filtering (BM3D) [45]. In this section, we summarize the main Neural Networks-based raindrop removal systems that have been developed in the last few years.

### 6.1. Dirt and Rain Noise Removal

#### 6.1.1. Overview

Eigen et al. [46] proposed a system to remove dirt and raindrop noise from a single image, using the NN architecture originally developed by LeCun et al. [47]. The system accepts a rainy image and generates a de-rained version of that image.

#### 6.1.2. Network Architecture

LeCun et al. [47] CNN define a multilayer network *y* = *F(x)* which is composed of “*L*” layers “*F*”, each applies a linear convolution to its inputs, followed by an element-wise sigmoid. For an *N* × *M* × 3 RGB input image,

$$F_{0}\left(x\right)\;=x$$

$$F_{l}\left(x\right)\;=\tanh\left(W_{l}*F_{l-1}\left(x\right)+b_{l}\right),$$

for l=1,..,L−1

$$F\left(x\right)\;=\frac{1}{m}\left(W_{L}*F_{L-1}\left(x\right)+b_{L}\right)$$

where “$W_{l}$” and “$b_{l}$” are the weigh and bias of network layers =1,..,L−1, respectively. “*m*” is the overlapping mask that accounts for kernel overlap near image boundary. Eigen et al. used two hidden layers, with 512 units in each layer and kernel size *P_l_* = 1 (1 × 1) per unit. The input and output layers have 512 units each, with kernels set to *P_1_* = 16 (16 × 16) and *P_L_* = (8 × 8), respectively. Stochastic Gradient Descent (SGD) is used to minimize the loss function:

$$J\left(\theta \right)\;=\frac{1}{2\left|D\right|}\underset{i\in D}{\sum }∥F\left(x_{i}\right)-y_{i}^{*}{∥}^{2}$$

#### 6.1.3. Baseline Methods and Training

Dataset $D=\left(x_{i},y_{i}^{*}\right)$, composed of (64 × 64) pixel subregions of clean and noisy images is used for training. For the rain dataset, pictures were taken of multiple scenes, with and without rain, where rain was simulated by spraying water on a pane of anti-reflective glass. For the dirt dataset, the opacity mask and additive component were first extracted from real dirt-on-glass panes. “Dirty” images were then simulated using the equation: $I^{\prime }=p\alpha D+\left(1-\alpha \right)I$, where “I” and “$I^{\prime }$” are the original and noisy image, respectively. “α” is a transparency mask and “*D”* being the additive component of dirt. “p” is a random perturbation vector in RGB space.

The system developed by Eigen et al. was compared against a multi-layer perceptron (MLP) system proposed by Burger et al. [44], a block-matching and 3D filtering (BM3D) system proposed by Dabov et al. [48], and against the median and bilateral filtering techniques.

For dirt noise, the system was trained using 5.8 million samples of (64 × 64) synthesized dirt noise, paired with a ground truth clear patch. Burger et al. Network was trained with 20 million (16 × 16) patches. For rain training, 6.5 million samples of size (64 × 64) each of synthesized rain noise were used.

#### 6.1.4 Experimental Results

- Synthetic dirt results showed that the proposed system and the MLP system outperformed the other three baseline methods. Testing with other types of sensitized noise, namely snow, and scratches on the image surface resulted in the system described by Eigen et al. producing near-zero PSNR (peak signal to noise ratio) which demonstrates that the system learned to remove dirt noise only.
- Real-life dirt results showed that the proposed system removed most dirt noise in the images, while the MLP left many more unremoved. The other three methods caused much degradation to non-dirt areas of the images. The proposed system failed to recognize large dirt noises and the ones that are out of shape, as well as dirt over areas that were not represented in the training set (e.g., bright orange cones).
- Synthesized rain that the proposed system outperformed the MLP system, in terms of removing more rain droplets and maintain non-rain areas of the image intact. The median filter needed to blur the image substantially before it was able to reduce raindrops.
- Real rain results showed that the proposed system successfully removed real raindrops from the captured images, but it started failing with the accumulation of water on the glass plate.

### 6.2 Attention GAN Raindrop Removal Algorithm

#### 6.2.1. Overview

Qian et al. [49] proposed a raindrop removal algorithm, based on an attentive Generative Adversarial Network (GAN). Their implementation of this algorithm is named “DeRaindrop” and it can be accessed through Git repository [1] algorithm Their work became the standard in GAN-based raindrop removal systems that researchers later try to improve upon. Their idea was to inject visual attention into the generative and discriminative networks during the training phase. This would push the generative network to pay more attention to the raindrop regions, and enabling the discriminative network to assess the local consistency of restored regions.

The generative adversarial loss of the system can be given by:

$$\underset{G}{\min}\;\underset{D}{\max}\;E_{R∼P_{clean}}\left[\log\left(D\left(R\right)\right)\right]+E_{R∼P_{raindrop}}\left[\log\left(1-D\left(G\left(I\right)\right)\right)\right]$$

where “G” and “D” represent generative and discriminative networks, respectively, “I” and “R” are the rain-degraded and clear images, respectively.

#### 6.2.2. Network Architecture

1- Generative Network

**Figure 21:**
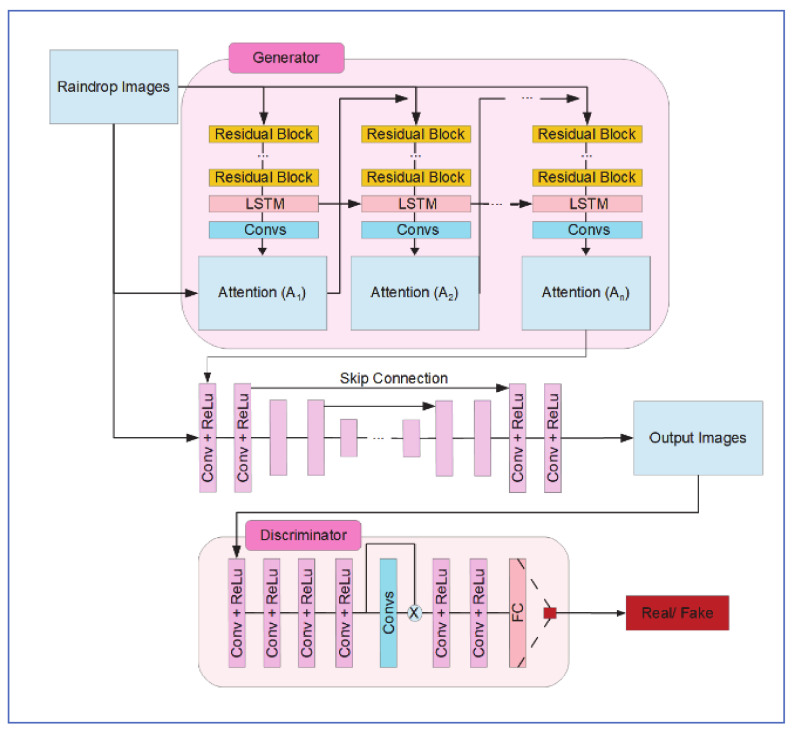
The architecture of Attentive GAN [49].

As shown in Figure 21, the generative network consists of two sub-networks; attentive-recurrent network and contextual autoencoder. The attentive-recurrent network uses a recurrent network of deep residual networks (ResNets) [50] combined with a convolutional LSTM [51], to generate an attention map of raindrops and surrounding structures. The output at each time step is a 2D attention map which is a matrix of numbers between 0 and 1, representing increasing attention from non-raindrop regions to raindrop regions.

The loss function of the attentive-recurrent network is given by:

$$L_{G}=10^{-2}L_{GAN}\left(O\right)\;+L_{ATT}\left(\left\{A\right\},M\right)\;+L_{M}\left(\left\{S\right\},\left\{T\right\}\right)+L_{p}\left(O,T\right)$$

where $L_{GAN}\left(O\right)\;=\log\left(1-D\left(O\right)\right)$, $L_{ATT}\left(\left\{A\right\},M\right)$ is the mean squared error (MSE) between the attention map A and binary mask M, $L_{M}\left(\left\{S\right\},\left\{T\right\}\right)$ is the multi-scale loss function, calculated as the MSE between the output $\left\{S\right\}\;$and ground truth $\left\{T\right\}$ sets, $L_{p}\left(O,T\right)$ is the Perceptual loss, calculated as the MSE between output image O  and ground truth T.

2- Discriminative Network

The discriminative network contains 7 convolution layers with a kernel of (3, 3), a fully connected layer of 1024 neurons, and a single-layer neuron with a sigmoid activation function, as shown in Figure 21.

The loss function of the discriminator is given by:

$$L_{D}\left(O,R,A_{N}\right)\;=-\log\left(D\left(R\right)\right)\;-\log\left(1-D\left(O\right)\right)\;+\gamma L_{map}\left(O,R,A_{N}\right)$$

where $L_{map}$ is the loss between features extracted from discriminator interior layers and the final attention map.

#### 6.2.3. Baseline Methods and Training

For training and testing, a data set of 1119 image pairs (clear, rainy) using SLR cameras, of outdoor scenes. Raindrops were synthesized by spraying a 3-mm-thick glass plate with water. The camera was set at a distance between 2 to 5 cm from the plate.

The system is compared to Pix2Pix system, described by Isola et al. [52], Eigen et al. system [46], and against three variants of the proposed system with lower content; contextual autoencoder alone (A), autoencoder plus discriminator (A + D), and autoencoder plus attentive discriminator (A + AD).

#### 6.2.4. Experimental Results

Table 2 below shows the comparison of the results from the proposed system against the two other CNN systems and the lower-content version of the system. The proposed system outperformed the other systems, both in terms of PSNR and SSIM (Structure SIMilarity index). Results also show the improvements in system performance with the help of attention maps (A + AD vs. A + D and A), where

A: contextual autoencoder alone,
A + D: autoencoder plus discriminator,
A + AD: autoencoder plus attentive discriminator.

### 6.3. Joint Shape-Channel Attention GAN Raindrop Removal Algorithm

#### 6.3.1. Overview

Quan et al. [53] realized that one issue with Qian et al. [49] approach is its dependence on the availability of high-quality raindrop masks for supervised training. They proposed an alternative approach that is unsupervised and makes use of a joint channel and shape-driven attentions to improve performance. For channel attention, Quan et al. introduced an attention/ recalibration mechanism in the CNN system, to enhance the contribution of more relevant features in the de-raining process versus less relevant ones. For the shape-driven attention, Quan et al. observed that the roundness of the raindrop shape can be approximated with an ellipse of a set of ellipses. Based on that, they developed a method to generate two edge maps, *B_1_* and *B_2_*, that identify raindrops in the input image. Starting with the simple linear model of rained image optical model [54],

$$I_{r}=\;\left(1-A\right)⊙L+A⊙R$$

where $I_{r},\;L,R\;\in R^{C\times M\times N}$ denote the rained image, latent raindrop-free layer, and the raindrop layer, respectively. $A\in {\left[0,\;1\right]}^{C\times M\times N\;}$ denotes the transparency matrix. The edge maps are concatenated with the rained image to create a tensor in $R^{\left(C+2\right)\times M\times N}$ which is later passed to the convolutional layer of the system. in this sense, the de-raining system proposed by Quan et al. implements a mapping $f:\left(I_{r},B_{1},B_{2}\;\right)\to I_{c}$, where $I_{c}$ is the resultant de-rained image [53].

#### 6.3.2. Network Architecture

**Figure 22:**
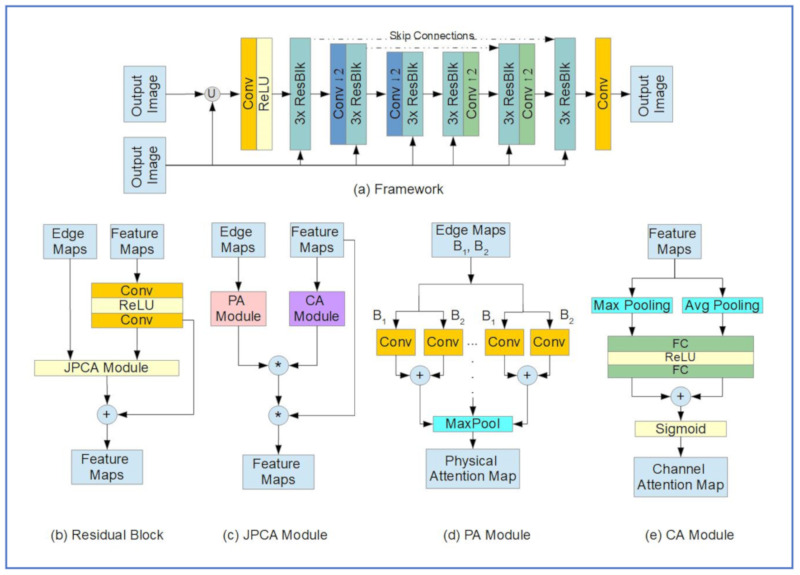
The Structure of the Joint Physical Shape/Channel Attention Raindrop Removal Algorithm (Quan et al. [53]).

Figure 22 below shows the main components of the rain removal algorithm proposed by Quan et al.

The system is based on the encoder-decoder topology and contains a convolution layer (Conv) with a rectified linear unit (ReLU) at the input side and a convolutional layer at the output. The encoder and decoder are arranged between the input and output layers, each made of nine residual blocks (Figure 22a). The Conv2 layers are added as down-sampling layers in the encoder and up-sampling layers in the decoder. Figure 22b,c show the inner arrangements of the residual block, joint physical-attention/channel attention (JPCA) module, physical-attention (PA) Module, and channel-attention (CA) module, respectively. A long skip connection is added in each residual block that connects it to the next block in the encoder/decoder setup. The loss function for training the network can be given by:

$$L=\frac{1}{N}\overset{N}{\underset{i=1}{\sum }}\|{\hat{X}}_{i}-X_{i}\|1$$

where *N* is the number of image pairs of de-rained “$\hat{X}$” and clear “X” images, and ‖.‖1 denotes the ${𝓁}_{1}$ distance [53].

#### 6.3.3. Baseline Methods and Training

Quan et al. used the same data set developed and published by Qian et al. [55] for training and testing their algorithm. For training, they used 861 image pairs that were 4x augmented, by applying horizontal flipping and resizing on the original images. For testing, 58 well-aligned pairs were used. Quantitative evaluation was done using PSNR and SSIM metrics and benchmarked against Qian et al. [55] and Eigen et al. [46] algorithms.

#### 6.3.4. Experimental Results

Quantitative results showed that the algorithm proposed by Quan et al. outperformed the other benchmarked algorithms in terms of PSNR and SSIM results, though it fell slightly behind Qian et al. algorithm for PSNR measurement of luminance. The ablation study showed that the JPCA module had the largest effect on improving system performance and that channel attention contribution was more noticeable than the physical shape attention module.

### 6.4. Improved Raindrop Removal with Synthetic Raindrop Supervised Learning

#### 6.4.1. Overview

One of the main issues for developing a good CNN-based raindrop removal system is the lack of training samples. It is generally quite hard to capture a large enough set of clear/rained of the same scene, necessary to train the CNN-based system. Hoa et al. [56] tried to solve this problem by developing a synthetic raindrop generator and using the synthetically generated rainy scenes, along with real raindrop real rainy scenes to train their GAN-based raindrop removal algorithm. Similar to the approach taken by Halimeh and Roser [12], Hoa et al. [56] based their raindrop synthesizer on ray-tracking to identify which points in the environment to use for rendering raindrops at specific locations on the windshield. This was followed by blurring and blending operations, to give synthesized raindrops more realistic optical perception.

#### 6.4.2. Network Architecture

Hoa et al. proposed raindrop removal network was composed of three sub-networks; a rain detection network, a reconstruction network and a refine network.

(a) Raindrop Detection Network

**Figure 23:**
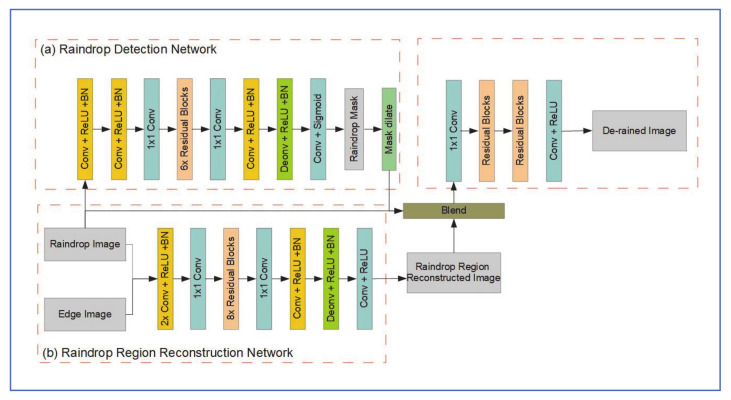
The Structure of the End-to-End Raindrop Removal CNN, proposed by Hoa et al. [56].

As shown in Figure 23a, the Detection network consists of 5 convolution layers and 6 residual blocks. All convolutional layers are followed by batch normalization (BN) and ReLU. This setup was inspired by the I-CNN structure that was proposed by Fan et al. [57], for general reflection removal and smoothing of images. The output of this network is a binary mask of the raindrops in the image, which is then dilated to reduce the number of raindrops in the mask and improve recall in later steps. Cross-entropy is used as the loss function which could be given by:

$$L_{det}\left(M,\hat{M}\right)=-\frac{1}{n}\overset{n}{\underset{i}{\sum }}\left[M_{i}\log\left({\hat{M}}_{i}\right)+\left(1-M_{i}\right)\log\left(1-{\hat{M}}_{i}\right)\right]$$

where $M,\hat{M}$ are the ground truth binary mask and the probability mask predicted by this network, respectively [56].

(b) Raindrop Region Reconstruction Network

As shown in Figure 23b, the reconstruction network is structured similarly to the detection network, with the residual blocks number increased from 6 to 8. The input image is combined with the edge image and used as 4-channel tensor input to the reconstruction network. ${𝓁}_{1}$ distance is selected as the loss function for this network.

(c) Refine Network

The original rained image R, the binary mask $\hat{M}$ from the detection network and the output of the reconstruction, network are blended as:

$$B=\hat{M}\hat{I}+\left(1-\hat{M}\right)R$$

where *B* is the blended image. This image is then passed to the refine network which, as shown in Figure 23, consists of two convolution layers and two residual blocks.

The loss function used in this network is a mix of SSIM loss and ${𝓁}_{1}$ distance, and is given by:

$$L_{ref}\left(I,\tilde{I}\right)=\alpha \left(1-L_{SSIM}\left(I,\tilde{I}\right)\right)+\left(1-\alpha \right)L_{𝓁1}\left(I,\tilde{I}\right)$$

where $\tilde{I}$ is the final output of the proposed algorithm [56].

#### 6.4.3. Baseline Methods and Training

Hoa et al. used their proposed raindrop synthesizer to generate a set of training and testing images, each image containing 50 to 70 raindrops. Images selected from the Cityscape dataset [58] were used as background. Thirty thousand images were used for testing and 1525 for testing the proposed algorithm. Further, online augmentation was added by randomly cropping segments from the input image or its horizontally flipped version. ADAM optimizer [59] was used for setting up the learning rate and epoch size of the networks. Using PSNR and SSIM as metrics, Hoa et al. benchmarked their proposed algorithm against the ones proposed by Eigen [46], Qian [49], as well as Pix2Pix [52], and DID-MDN [60] algorithms that were not designed originally for adherent raindrop removal. These different algorithms were trained using image pairs of clear and synthetically generated raindrops.

#### 6.4.4. Experimental Results

The quantitative results showed that Hoa et al. proposed algorithm performed better than all other algorithms in terms of SSIM and PSNR scores. Qualitatively, the proposed algorithm, as well as Qian’s and the DIID-MDN removed most raindrops successfully. The proposed algorithm as well generated de-rained images with the least artifacts, as compared to other methods benchmarked. Additionally, the network was trained using a dataset published by Qian et al. [49], with only a rough mask used for training, due to the lack of a high-quality raindrop mask. Results showed that the proposed algorithm produced results that were on-par with Qian [49] and better than all other methods.

### 6.5. Raindrop-Aware GAN for Coastal Video Enhancement

#### 6.5.1. Overview

Nearshore wave process is an essential problem in coastal studies and CNN-based systems can help in the process of learning coastal wave behavior. Adverse weather conditions, especially rain, degrades the quality of captured images/videos which makes them difficult to use as training data [61]. Kim et al. [61] proposed an algorithm for unsupervised learning with (GAN)-based video generation (Raindrop-aware GAN) to enhance a raindrop-contaminated coastal video. The algorithm was built on an encoder/decoder architecture, dilated convolution blocks, and long skip connections. A new loss function was also introduced to guide the adversarial.

#### 6.5.2. Network Architecture

**Figure 24:**
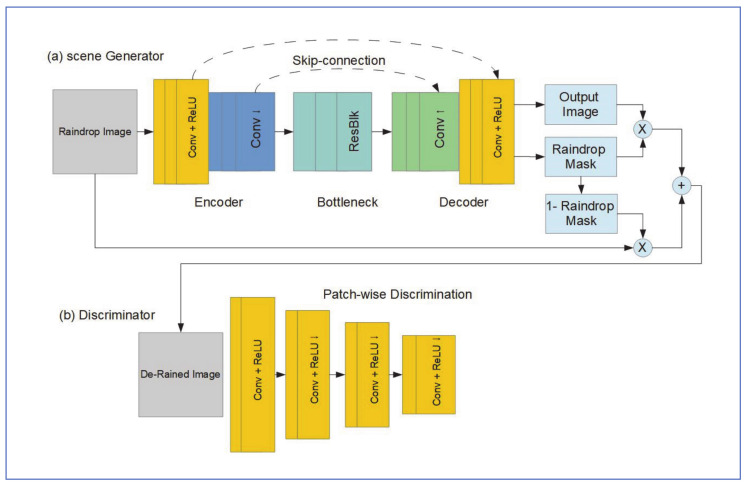
Structure Raindrop-Aware GAN raindrop removal network, as proposed by Kim et al. [61].

As shown in Figure 24, the CNN structure proposed by Kim et al. [61] consists of two sub-networks, the scene generator network, and the discriminator network. The generator network attentively restores the rained image background guided by the raindrop mask, while the adversarial learning tries to restore the natural appearance of the coastal waves in the background. The process continues until the discriminator is deceived to believe restored rainy images belonged to the clear image set [61].

(a) Scene Generator

The encoder of scene generator, as shown in Figure 24a, is made of collections of convolution layers, followed by ReLU units. The convolution layers are set to apply downsampling of the input image. The decoder structure is similar to that of the encoder but with upsampling rather than downsampling. The bottleneck section is made of residual blocks, and it improves the robustness of the detection of raindrop regions of different sizes. The added skip-connections deliver early layer features of the encoder to the decoder [61].

Three loss functions are minimized in the scene generator network, $L_{gen,\;},L_{mask},\;\mathrm{and}\;L_{reg}$. $L_{gen,\;}$ is used to train the scene generator to deceive the discriminator, and can be given by:

$$L_{gen,\;}=E_{r_{i\in }I_{rain}}\left[\|D\left({\bar{r}}_{i}\right)-{1\|}_{2}^{2}\right]$$

where $r_{i}\;$and ${\bar{r}}_{i}$ and the original rained image and resorted image of sample *i*, respectively, and $D\left({\bar{r}}_{i}\right)$ is the output of the discriminator of that image sample. $I_{rain}$ represents the set of training instances of distorted images. $L_{mask}$ is implemented as ${𝓁}_{1}$ distance loss function and it helps the generator focus on rain regions while preventing the mask from saturation [61]. $L_{reg}$ represents the regularization loss of a randomly selected clean image ($c_{j}$) from the training set. It can be given by:

$$L_{reg}=E_{c_{j\in }I_{clean}}\left[\|\left(1-m_{j}^{c}\right)⊙o_{j}^{c}-c_{j}\|1\right]$$

where $m_{j}^{c}$ is the mask, $o_{j}^{c}$ is the restored image and $I_{clean}$ is the training set of clean images. That total loss LG of the scene generator is given by:

$$L_{G}=w_{1}L_{reg}+w_{2}L_{mask}+w_{31}L_{gen}$$

where $w_{1}$,$\;w_{2},\;and\;w_{3}$ are hyperparameters to control the importance of each loss function [61].

(b) The Discriminator

The discriminator network was constructed in the PatchGAN architecture, originally proposed by Isola et al. [52]. It is constructed from a series of convolution layers, each followed by a ReLU activation function. The series of layers downsample the input to the discriminator, resulting in an output size of w/32 X h/32 X 1. The loss function implemented for the discriminator is given by:

$$L_{D,\;}=E_{r_{i\in }I_{rain}}\left[\|D{\left({\bar{r}}_{i}\right)\|}_{2}^{2}\right]+E_{c_{j\in }I_{clean}}\left[\|D\left(c_{j}\right)-{1\|}_{2}^{2}\right]$$

#### 6.5.3. Baseline Methods and Training

For training and testing, Kim et al. used the dataset published by Qian et al. [49], as well as two datasets generated by capturing videos and still image pairs of Ankmok Beach. The proposed network was initialized by He et al. initialization [62] and training by ADAM optimizer [59]. PSNR, SSIM, and natural image quality evaluator (NIQE) [63] were used to benchmark the performance of the proposed algorithm against Pix2Pix [52] and Attentive GAN [49] algorithms.

#### 6.5.4. Experimental Results

Using Qian et al. [49] dataset for training and testing showed that both attentive GAN [49] and Kim et al. proposed algorithm improved quantitative score of PSNR and SSIM of restored image versus distorted one. The three algorithms were then retrained and tested using Anmok paired image dataset. Attentive GAN performed poorly and scoring worse on the restored image versus distorted on for all three metrics. Pix2Pix performance was even worse than Attentive GAN in a quantitative measure of performance. Kim et al. proposed algorithm performed best, improving scores on all three metrics between distorted and restored images. The proposed method again outperformed the two other algorithms, when an unpaired image dataset was used for training and testing. Only the NIQE metric was used since the images were not paired.

### 6.6. Self-Supervised Attention Maps with Spatio-Temporal GAN

#### 6.6.1. Overview

Alletto et al. [64] realized the limitation in the dataset created by Qian et al. [49] both in terms of size and variety of raindrop images. They proposed a rainy image syntonizer that superimposes photo-realistic raindrops on real images. Additionally, they proposed a rain removal system that is done in two stages. The first stage is a single-image rain removal network that removes raindrops from the first few frames of a sequence. The second stage is a Spatio-temporal network with optical flow explicitly integrated into the network. The raindrop synthesizer employed to commonly screen-space effects commonly used in games, namely Refraction and depth-of-field. A refraction direction buffer is first generated by ray-tracking and mapping each raindrop pixel to the environment points involved in rendering the raindrop. A second buffer is then generated that contains the background image from the first buffer. Finally, a disk of confusion is added to generated raindrops through applying defocus blur and Bokeh effect [65]. A parameter is added to the bokeh effect to allow it to be set anywhere from fully focused up to fully defocused.

#### 6.6.2. Network Architecture

(a) Single-Image Removal

The proposed rain removal system begins with a single-image unsupervised CNN. This network is made up of two parts; location map estimator and raindrop remover. Encoder/decoder structure is selected to construct both parts of the single-image network. In the first part, the system learns in an unsupervised manner the location of potential raindrops in the rainy image. The system tries to minimize the loss function given by:

$$L_{A}\left(R,R^{\prime }\right)\;=\|R-{R^{\prime }\|}_{2}^{2}$$

where *R* is the rainy image and $R^{\prime }=\frac{C+G_{A}\left(R\right)}{2}$ is the reconstructed rainy image obtained by averaging the clean image *C* with the additive location map outputted from the generator network, $G_{A}\left(R\right).$ The location map (mask of raindrops) *A* is then fed to the next part of the network, along with the rainy image *R*. the location map estimator and rain remover are jointly trained to minimize the final objective function:

$$L\left(R,R^{\prime },C,C^{\prime }\right)\;=\alpha L_{A}\left(R,R^{\prime }\right)\;+\alpha L_{VGG}\left(C,C^{\prime }\right)\;+\beta L_{Adv}\left(R,R^{\prime }\right)\;+\beta L_{adv}\left(C,C^{\prime }\right)$$

where $L_{VGG}\;$ is the VGG perceptual loss and $L_{adv}$ is the adversarial loss. α and β are hyperparameters that are selected empirically.

(b) Spatio-Temporal Raindrop Removal

**Figure 25:**
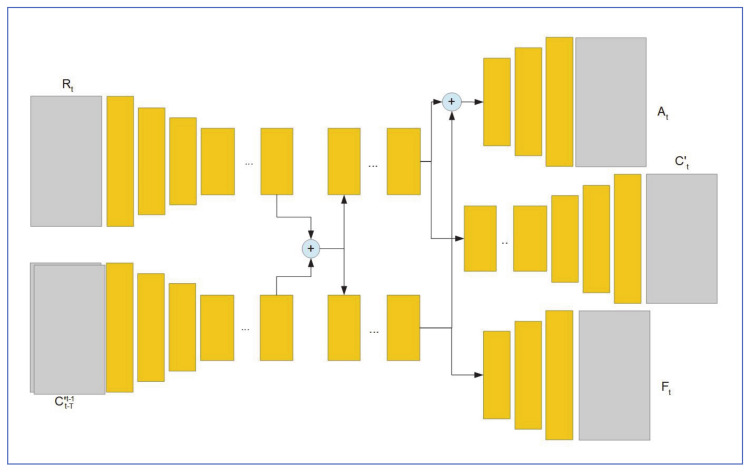
Structure of Spatio-Temporal GAN raindrop remover, as proposed by Alletto et al. [64].

Figure 25 shows the complete rain removal system as proposed by Alletto et al. the network is structured as encoder/decoder architecture, where the encoder branches (upper left two in Figure 25) extract features from rainy image Rt and the previous T-1 restored images, $C\prime_{t-T}^{t-1}$. The resulting feature maps are concatenated and fed to an additive map and an optical flow map. The map estimated feature maps are concatenated with flow features and then fed to the location estimator section (upper right section in Figure 25). For optical flow, the optical flow estimator system aims to optimize the following objectives:

$$L_{W}=\|C_{t}-W_{t}\|{}_{2}^{2}$$

$$L_{FAdv}=E\left[logD_{f}\left(\tilde{F_{t}}\right)\right]+E\left[log\left(1-D_{f}\left(F_{t}\right)\right)\right]$$

where $W_{t}$ is the result of wrapping optical flow $F_{t}$ with $C_{t-2}$ previously stored image frames. $D_{f}$ is the discriminator part of the optical flow estimator.

#### 6.6.3. Baseline Methods and Training

Attello et al. augmented videos from DR(eye)VE dataset [66] with synthetically generated raindrops. Sequences with cloudy backgrounds were selected since they provided the most realistic rainy weather scenes. Qian et al. [49] dataset was also used for the single-image performance assessment. PSNR and SSIM quality metrics were used to benchmark the proposed algorithm against Qian et al. [49], Eigen et al. [46], and Pix2Pix [52] algorithms.

#### 6.6.4. Experimental Results

Results showed that the proposed algorithm outperformed the benchmarked ones for the single-image de-raining task. For the DR(eye)VE testing, Vid2Vid [67] and a rain-streak removal [68] algorithms were used instead of the Pix2Pix and Eigen et al. algorithms. Results again showed that the proposed algorithm overperformed the other algorithms, both in the PSNR and SSIM metrics. For temporal consistency evaluation, two measures were used. A Human Performance Score (HPS) was calculated from the feedback of human participants on the visual quality of the outputs. Alletto et al. proposed algorithm output was the preferred one among all evaluated ones. On the second test, Fréchet Inception Distance (FID) [69] was used to measure the temporal consistency of de-rained to clean videos. Results showed that the proposed algorithm performed best and Vid2Vid performed worst.

### 6.7. Concurrent Channel-Spatial Attention and Long-Short Skip Connections

#### 6.7.1. Overview

Peng et al. [70] proposed a CNN-based system for raindrop removal that employs concurrent channel and spatial attention. While channel attention allows the system to focus on more relevant features in the rain removal and image restoration process, spatial attention allows for different treatments of regions of the image with different levels of rain distortion. The proposed solution was built on the encoder/decoder architecture, with added long skip-connections between the encoder and the decoder units, and short skip-connections to link layers inside the encoder and encoder blocks. Channel squeeze-excitation (SE) attention blocks were added to the CNN to reweight feature channels by exploiting interdependencies between the channels. Special attention SE blocks were added to generate spatial attention masks that emphasized severely degraded image regions due to rain versus lightly degraded ones (e.g., mist) [70]. The proposed approach in many aspects is similar to the one proposed by Quan et al. [53] who also employed channel and special attention mechanism and used short and long skip-connections. Peng et al., however, implemented their solution differently, which will be shown below.

#### 6.7.2. Network Architecture

(a) Overview

**Figure 26:**
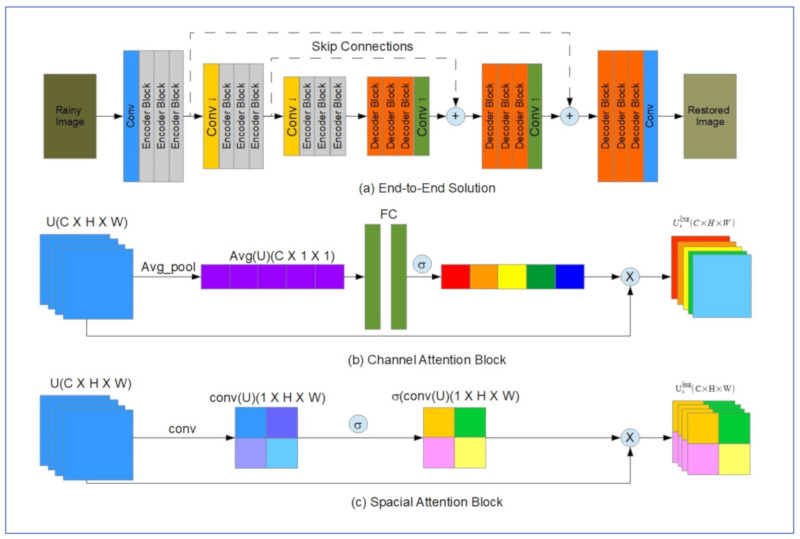
Raindrop removal system with concurrent spatial and channel attention, as proposed by Peng et al. [70].

As shown in Figure 26a, the encoder and decoder blocks have a similar structure. Each encoder group connects a down-sampling layer with three convolution layers. The decoder groups each have one up-sampling layer connected to three convolution layers. Long and short skip-connections are shown to connect encoder/decoder blocks at different stages.

(b) Channel Attention

Figure 26b shows the structure of the channel attention block. Starting with a set of feature maps {*U*_1_, *U*_2_,…, *U_c_*} of C channels, a special squeeze is applied on this set by computing a vector ***a*** = [*a*_1_, *a*_2_,.., *a_c_*], that represents the global average of the individual feature channel maps, *U*_1–_*_c_*. channel excitation is done by passing the vector ***a*** to a two-layer MLP which outputs an output vector ***p*** = [*p*_1_, *p*_2_,…, *p_c_*]. The values of elements in p range from [0, 1], representing the importance of each feature channel. The recalibrated feature map can now be calculated as:

$${\hat{U}}_{C}^{CSE}=P_{C}U_{C}\;\forall C$$

(c) Spatial Attention

Figure 26c shows the structure of the spatial attention block. Starting with a feature map *U*, a spatial squeeze is applied using a 1 × 1 convolution over *U*. The output of convolution, *B*, is passed to a sigmoid excitation function which results in a normalized version of *B*, where each element of the new map,$\;Q\left(i,j\right)\in \left[0,\;1\right]$ for any location (*i*, *j*) in the image. *Q* represents the spatial significance of the feature maps and can be used to recalibrate the feature maps spatially as follows, [70]:

$${\hat{U}}_{C}^{SSE}=B\left(i,j\right).U_{c}\left(i,j\right)\;\forall C$$

(d) Concurrent Attention

The concurrent channel and spatial attention can be given by:

$${\hat{U}}_{C}={\hat{U}}_{C}^{CSE}+{\hat{U}}_{C}^{SSE}\;\;\;\;\;\forall C$$

For system optimization, Peng et al. proposed to minimize the loss function given by:

$$\underset{\Omega }{\min}\underset{i=1}{\sum }\|f\left(R_{i};\;\Omega \right)\;-C_{i}\|{}_{2}+\lambda \|E\left(f\left(R_{i};\;\Omega \right)\right)\;-E{\left(C_{i}\right)\|}_{2}$$

where $f\left(R_{i};\;\Omega \right)$ represents the CNN function that maps rainy image *R* to clear output, according to network parameters stored in Ω. *E*(.) represents the output of the encoder. The first term optimizes the global approximation of the recovered image to the true clear image. The second term guides the recovery of image features adaptively [70].

#### 6.7.3. Baseline Methods and Training

Peng et al. used Qian et al. [49] dataset for training and testing their proposed solution. For quantitative evaluation, they used PSNR and SSIM metrics to benchmark the performance of their proposed system against Eigen [46], Pix2Pix [52], Qian [49], PreNet [71], and DDN [72], algorithms.

#### 6.7.4. Experimental Results

Experimental results showed that Peng et al. proposed solution outperformed Pix2Pix, DNN, PreNet, and Eigen algorithms by a big margin. The proposed solution also slightly outperformed Qian’s algorithm with the added value of smaller system size. An ablation study to verify the contribution of each system block to the overall performance showed that the concurrent attention module had a big contribution to improving recovered image visual quality.

### 6.8. Separation-Restoration-Fusion Network for Image Raindrop Removal

#### 6.8.1. Overview

Ren et al. [73] proposed a raindrop removal system that was based on the proven “divide and concur” approach to solving complex problems. The process starts with separating the rainy image into different segments based on the amount of image distortion caused by raindrops. Each segment then is optimized individually to achieve the best image restoration results. The restored segments finally are fused to construct the full recovered image. The rainy image model per Ren et al. can then be given as:

$I=I_{r\_l}+I_{r\_h}+I_{f}$, where $I_{r\_l},I_{r\_h}\;and\;I_{f}$ represent lightly damaged, heavily damaged, and free region of the rainy image.

#### 6.8.2. Network Architecture

(a) Region Separation Module

**Figure 27:**
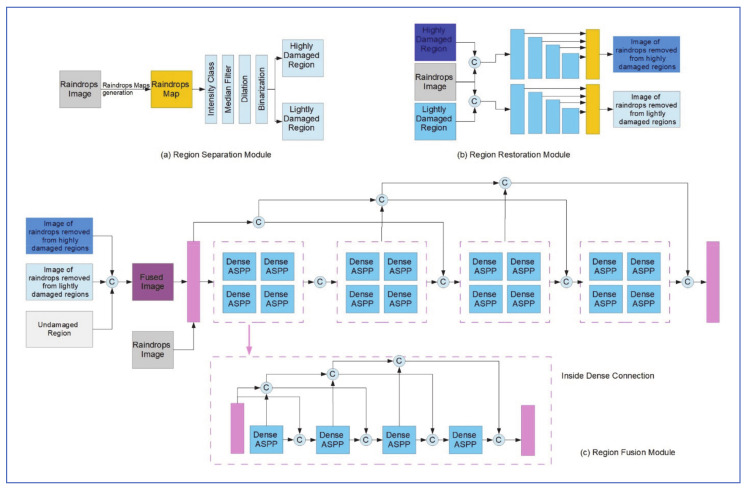
Structure of raindrop removal system with Separation-Restoration-Fusion approach, as proposed by Ren et al. [73].

As shown in Figure 27a, the separation stage starts with creating a raindrop map that may contain elements other than raindrops. Then, pixel-level classification is applied on the map to map each pixel to either the heavy or light distortion region, based on its intensity. This is followed by a median filter to reduce noise and dilation to smooth the edges. The separation process concludes by applying image binarization on the filtered and diluted mask, to generate Highly damaged and lightly damaged regions [73].

(b) Region Restoration Module

As shown in Figure 27b, the original rainy image is individually fused with both highly damaged and lightly damaged regions from the previous separation network. A multi-scale feature fusion GAN (MFGAN) network is used which is built on a five-scale pyramid structure. A local spatial module that is based on squeeze-excitation block is added, to avoid noisy information from shallow layers and guide the network to pay more attention to damaged regions.

(c) Region Fusion Module

To fuse the individual regions back into one recovered image, Ren et al. developed a new connection scheme, based on the DenseASPP module [74]. This connection is called inside and outside dense connection network (IODNet). As shown in Figure 27c, this network makes use of 16 DenseASPP modules, divided into 4 groups. IODNet enhances the flow of information within each DenseASPP group first through a sequence of concatenations of the outputs of each DenseASPP with its neighbors. the groups are then densely concatenated together to provide the “out” part of the IODNet.

For loss function, Ren et al. used the smooth MAE [75] loss with low sensitivity to outliers was used as a measure of closeness of output to the input image. The loss function can be given by:

$$L_{smooth}=\frac{1}{N}\overset{N}{\underset{x=1}{\sum }}\overset{3}{\underset{i=1}{\sum }}F_{smooth}\left({\hat{J}}_{i}\left(x\right)-J_{i}\left(x\right)\right)$$

where ${\hat{J}}_{i}\left(x\right)$ denotes the ith color channel of pixel *x* in the output image and *N* is the total number of pixels. $F_{smooth}$ can be given by:

$$F_{smooth}\left(e\right)=\left\{\begin{array}{c} 0.5e^{2},\;if\;\left|e\right|<1\\ \left|e\right|-0.5,\;otherwise \end{array}\right.$$

The objective function of the network is given by:

$$L=\lambda_{r}L_{r}+\lambda_{out}L_{out}$$

where $L_{r}$ is the sum of smooth MAE between the restored image regions and target region images, and $L_{out}$ is ${𝓁}_{1}$ *distance loss* between regions-fused image and the target image. $\lambda_{r}\;\mathrm{and}\;\lambda_{out}\;$are determined experimentally [73].

#### 6.8.3. Baseline Methods and Training

Ren et al. used the Qian et al. [49] dataset to train and test their network. ADAM optimizer [59] was used to set batch size, learning rate, and decay rates. Randomly chopped image segments were used as input/truth data. PSNR and SSIM quality metrics were used to benchmark the proposed algorithm against Qian et al. [49], Eigen et al. [46], Li et al. [76], Quan et al. [53], and Pix2Pix [52] algorithms.

#### 6.8.4. Experimental Results

Experimental results showed that Ren et al. proposed system outperformed all other methods that were benchmarked, including the newer approaches proposed by Quan et al. [53] and Li et al. [76]. The improved performance was especially visible on an image with complex backgrounds. Focusing on rain-free regions, Ren et al. observed that their proposed system does not distort rain-free regions of the image. As a comparison, Quan et al. [53] approach added noise and changes the color of some rain-free segments. Qian et al. [49] made the rain-free region blurred. An ablation study showed that all components of the system provide a valuable contribution to the performance of the system.

## 7. Summary

In this section, we summarize in tabular format, the most important aspects of the adherent raindrop removal systems that were described in this survey paper. Table 3 lists the different raindrop models that were described in this paper. Table 4 summarizes the common classical approaches for raindrop detection. A list of different de-raining techniques is shown in Table 5. Table 6 shows a comparison of the Deep Learning and CNN approach to image de-raining that were described in this survey paper.

## 8. Conclusions

We described a range of research works in the field of adherent raindrop detection and removal, with a focus on applications in the automotive domain. Based on the reviewed research work in this paper, we conclude the following:

1- Adherent raindrop detection and removal is a more challenging problem than falling rain detection and removal, due to the persistence of adherent raindrops over many image frames and the irregularity of raindrop shapes and sizes.
2- Due to the closeness to the image plane, adherent raindrops look blurry and occlude larger areas of the captured image.
3- Due to the above, most reviewed algorithms performed poorly under heavy rain conditions or fast-changing scenes with many moving objects.
4- Simple detection algorithms were based on observed optical or physical characteristics of adherent raindrops and performed well if the presumed conditions were met. Performance is degraded quickly for any deviation from these conditions, including change of background image texture or illumination and the introduction of moving objects in the scene background.
5- Complex detection algorithms performed very well under low and medium rain conditions. The added complexity, however, can introduce unacceptable latencies in real-time applications for processing rained images and removing adherent rain.
6- Compromises were discussed to improve processing time that included limiting the ROI, reducing the number of model templates, and dimension reduction, among other things.
7- The application of Deep-learning and CNN seems to be a very promising approach for solving the raindrop detection and rain removal problems.
8- The use of PSNR and SSIM metrics may not be the best choice for performance evaluation and benchmarking among different CNN-based algorithms. Results reported by different researchers showed marginal improvement in PSNR and SSIM scores which may very much be within the statistical margin of error.

## Figures and Tables

**Table 1:** Truth Table to Distinguish True Raindrop from Background Noise.

Texture Complexity	Intensity Variance (Edge)	Intensity Variance (Inner)	Type
Simple	Large	Small	Raindrop
	Small	Large	Background
Rich	Large	Small	Raindrop
	Large	Large	Background

**Table 2:** Quantitative Evaluation Results of System Proposed by Qian et al. [49] Against other Systems.

Method	Metric	
	PSNR	SSIM
Eigen [46]	28.59	0.6726
Pix2Pix [52]	30.14	0.8299
A	29.25	0.7853
A + D	30.88	0.8670
A + AD	30.60	0.8710
AA + AD	31.57	0.9023

**Table 3:** Raindrop Models.

ID	Basic Idea	Potential Limitations
Cord et al. [38]	Assume elliptical shape for raindrops and use axes aspect ratio, size, and brightness constraints as a model for raindrops.	It May not account for irregular raindrop shapes and the effect of background texture on raindrop appearance.
Kurihata et al. [18]	Used a PCA algorithm to generate eigendrop templates.	Does not account for the effect of texture on raindrop appearance.
Fouad et al. [11]	Use a declivity operator to describe raindrops as a sequence of peaks and valleys.	Do not consider the background composition role in the appearance of raindrops.
Halimeh et al. [12]	Developed a complex model (RIGSEC) for a raindrop, based on its geometric and photometric properties.	Assuming part of a sphere for a raindrop and ignoring the blurring effect of a raindrop may limit model accuracy.
Roser et al. [21]	Added blurriness effect to RIGSEC and limited the rendering of raindrop models to certain regions of the image to reduce rendering time.	Generating raindrop models at specific regions in the image may lower the rate of matching with real raindrops.
Sugimoto et al. [43]	Used MSER to improve the initial detection of potential raindrops and spheroid for raindrop approximation.	Added complexity may make the model less appropriate for real-time applications.
Stuppacher et al. [22]	Modeled raindrops using height maps, considering raindrop dynamics and water content losses and gains for moving raindrops.	The model is more suitable for CGI applications to generate realistic raindrop effects.
Roser et al. [24]	Modeled raindrops using Bézier Curves.	Reliance on approximations of raindrop size from correlations between 2D ratios and tilt angles reduces model accuracy.

**Table 4:** Raindrop Detection-Classical Approaches.

ID	Application	Approach	Potential Limitations
Yan et al. [14]	Weather classification in the automotive domain	Use AdaBoost to combine two weak classifiers, HGA and HSV. Classifies weather as Rainy, Cloudy or Sunny	Applications of weather classifiers are limited in the automotive domain to ADAS warnings and windshield wiper triggers.
Wu et al. [35]	Raindrop detection in the automotive domain	Use AdaBoost to combine color, shape, and texture saliency maps. Create a raindrop visual descriptor and use SVM to classify the weather.	Assumes circular 2D shape of a raindrop and fails under heavy rain conditions
Liao et al. [36]	Raindrop detection in the automotive domain	Segment the scene into the roadway and building areas. Identify raindrop candidates through edge detection and binarization and compare their dimensions to the closest ellipse.	The detection algorithm might be slow for real-time automotive applications and it fails to handle background noise and large raindrops.
Ishizuka et al. [37]	Raindrop detection in the automotive domain	Daytime Detector uses Sobel for initial identification, then texture information, and optical flow to detect real raindrop pixels. Nighttime Detector eliminates light source pixels, then uses a temporal intensity change feature to identify raindrop pixels.	The optical flow approach used assumes straight-line driving and may fail on winding roads. It may also cause incorrect classification as raindrops, objects that are moving at the same speed as the test vehicle. (e.g., other vehicles).
Kurihata et al. [18]	Raindrop detection in the automotive domain	Use similarity degree between potential raindrops and eigendrop template to identify raindrop regions	Does not account for the effect of background variations on raindrop characteristics (texture, brightness).
Yamashita et al. [28]	Raindrop detection in surveillance applications	Match images from different cameras, then analyze intensity variance under low and high texture image background to detect raindrops.	Requires multiple cameras which reduces the common FOV, and assumes raindrops do not occlude the same section of the restored image
Yamashita et al. [29]	Raindrop detection in surveillance applications	Capture successive image frames and identify them as raindrop segments, those that are detected near the expected location and satisfy size ratio constraint.	Requires precise knowledge of rotation angle and assumes idle raindrops between frames which is true only under light rain conditions.
Yamashita et al. [30]	Raindrop detection in surveillance applications	Similar to [29] but rotation angle is estimated and raindrop decision is made on a pixel base, by measuring the noise existence rates in the original and rotated image.	Assumes idle raindrops between frames which is true only under light rain conditions.
Yamashita et al. [31]	Raindrop detection in surveillance applications	Match stereo image pixels using NCC and apply one-on-one matching to eliminate noise. Compare measured to the expected disparity of raindrops to determine true raindrops.	Raindrops are blurry and may not result in good disparity measurements. Additionally, the long computational time is observed as a result of pixel-based calculations.
Yamashita et al. [33]	Raindrop detection in surveillance applications	Create a compound image from the temporal image sequence and select raindrop pixels that show “often” in the noise candidate trajectory curve.	Requires many frames and involves many pixel projections.
Roser et al. [16]	Weather classification in the automotive domain	Use feature histogram to create a bag of features, and use SVM to classify weather as Clear, Light rain, or Heavy rain.	Relatively slow, due to the large descriptor. Error rate increases with background complexity increase.
Cord et al. [17]	Weather classification in the automotive domain	Compare the intensity gradient image to the threshold image and pick the strongest candidates. Pick raindrop regions based on dimensions and eccentricity constraints and through temporal analysis.	System Requires focused raindrops (camera needs to be attached far away from the windshield). Raindrop size is relatively small (3–10 pixels) which may cause reduced accuracy.
Cord et al. [38]	Raindrop detection in the automotive domain	Segment the image then uses either watershed or background subtraction to identify potential drops. Use size, shape, and temporal constraints to identify real raindrops.	The algorithm runs slow due to implementation in MATLAB. Adding more frames improves performance but adds delay to the overall system operation time.
Nashashibi et al. [40]	Raindrop detection in the automotive domain	Detect potential raindrops through temporal intensity change and shape roundness. Use a lack of clear contour as a raindrop characteristic, then verify selection by spatially matching raindrop regions in consecutive frames.	Detection of unfocused is challenging and the algorithm fails under bright background conditions.

**Table 5:** Raindrop-Degraded Image Recovery.

ID	Approach	Potential Limitations
Liao et al. [36]	For buildings ROI, replace the raindrop area with the closest non-rain area (using an 8-connected area template). For road ROI, use inpainting or morphological operations.	Removal time is long (0.44 to 0.68 s per frame) and it is proportional to rain density. The restoration of the road mark sections of the image is not perfect, due to the limitations of the inpainting method.
Wu et al. [35]	Use the inpainting technique through smooth propagation in the equal intensity line direction.	Limited to low and medium rain intensity. Inpainting based on intensity does not preserve or recover the textural characteristics of the recovered regions.
Yamashita et al. [29]	Create a composite image of the original and rotated one, with a parameter that controls how much each image is contributing to the final composite one.	Chromatic variations between original and rotated images may still exist, even with correction. This affects the quality of the recovered image. The algorithm fails if the difference between original and rotated images is large.
Yamashita et al. [33]	Decompose the image into structure and texture images. Apply inpainting process on structure image and texture synthesis process on the other.	The Spatio-temporal analysis may be needed to improve texture recovery but this, in turn, may add delay to the processing time.
Yamashita et al. [31]	Use disparity information to identify proper regions from the complementary image in the pair for raindrop pixel substitution.	Relies on imperfect disparity map data to select substation pixels. Additionally, the approach fails if raindrop noise is present in both images.
Roser et al. [21]	Estimate translational and rotational parameter vector *h* probabilistically, then use multi-band blending to recover rained regions.	While producing good results, this algorithm, both in its detection and recovery section seems to be too computationally expensive for automotive applications.

**Table 6:** Deep Learning and CNN Approach to Image De-Raining.

Raindrop Removal System	Network Architecture	Datasets and Testing	Potential Limitations
Dirt and Rain Noise Removal (Eigen et al. [46])	multilayer convolutional network with two hidden layers with 512 units each.	Pictures of a glass plate with dirt and water drops were taken. Patches of size 64 × 64 for dirt and were paired with clear patches and used to train and test the rain and dirt remover system.	Restored images showed visible artifacts and were blurred where the raindrop/dirt particle was removed.
Attention GAN Raindrop Removal Algorithm (Qian et al. [49])	Generative Network: Attention Map (3 layers of ResNet + 1 LSTM) Autoencoder (16 conv-ReLU with skip connection). Discriminative Network: 7 convolution layers with the kernel size (3 × 3), a fully connected layer of 1024 neurons, and a single neuron with a sigmoid activation function	1119 pairs of images (rainy and clear), with various background scenes and raindrops. Raindrops are synthesized by spraying water on a glass plate.	Limited dataset for training and testing. Need for raindrop mask for supervised learning
Joint Shape-Channel Attention GAN Raindrop Removal Algorithm (Quan et al. [53])	GAN-based on encoder-decoder architecture with ResBlocks in between and short and long skip connections. Joint physical and channel attention blocks	Used Qian et al. [49] dataset for training and testing. Uses PSNR and SSIM were used for evaluation and benchmarking.	Dataset limitations for training and testing. PSNR and SSIM scores were only marginally better than other algorithms evaluated.
Improved Raindrop Removal with Synthetic Raindrop Supervised Learning (Hoa et al. [56])	The system consists of three sub-networks rain detection network, (I-CNNN with 5 conv layers + BN and ReLU activation, and 6 Resblocks reconstruction network, same as detection network with 8 ResBlocks refine network, 2 conv layers, and 2 ResBlocks	Synthesized rainy images and used them for training and testing. Adam optimizer was used for setting up training parameters. PSNR and SSIM were used for evaluation and benchmarking.	The quality of images generated by the Rain Synthesized needs more independent evaluation against real rainy images.
Raindrop-Aware GAN for Coastal Video Enhancement (Kim et al. [61])	Encoder/decoder architecture with short and long skip connections.	Used Qian et al. [49] dataset as well as Anmok beach videos and paired image sets for training and testing. PSNR, NIQE, and SSIM were used for evaluation and benchmarking.	Though outperforming other methods in the coastal setup, for urban setup no clear improvement was observed over Qian et al. [49].
Self-Supervised Attention Maps with Spatio-Temporal GAN (Alletto et al. [64])	The system is made of two-stages. 1. Single-Image Removal, with location map estimator and raindrop remover networks. both constructed based on encoder/decoder architecture. 2. Spatio-temporal Raindrop Removal, flow estimator provides optical flow is learned from previous frames and concatenated with rainy image and estimated location map in a decoder/ encoder GAN architecture.	Used Qian et al. [49] dataset as well as data set of augmented videos from DR(eye)VE dataset with synthetically generated raindrops. PSNR and SSIM were used for evaluation and benchmarking.	The quality of images generated by the Rain Synthesized needs more independent evaluation against real rainy images.
Concurrent channel-spatial attention and long-short skip connections (Peng et al. [70])	The system was built on the encoder/ decoder architecture, with channel and spatial attention blocks added. Short and long connections were also introduced.	Used Qian et al. [49] dataset for training and testing. Uses PSNR and SSIM were used for evaluation and benchmarking.	Dataset limitations for training and testing. The approach is similar to Quan et al. [53] with differences in network architecture. Would be interesting to compare the performance of one against the other.
Separation-Restoration-Fusion Network for Image Raindrop Removal (Ren et al. [73])	the system consists of three modules. 1. Region separation module was implemented as an image pipeline with classical techniques 2. Region restoration module MFGAN built on pyramid topology was used. 3. region Fusion module IODNet connection network using DenseASPP was used to construct fusing module	Used Qian et al. [49] dataset for training and testing. Uses PSNR and SSIM were used for evaluation and benchmarking.	Images need preprocessing to classify regions of the image based on the severity of the raindrop. The classification was based on experimental results from a limited dataset and may not apply to other scenarios.

## Data Availability

Not applicable.
